# Single cell transcriptome analysis of developing arcuate nucleus neurons uncovers their key developmental regulators

**DOI:** 10.1038/s41467-019-11667-y

**Published:** 2019-08-16

**Authors:** Christian Huisman, Hyeyoung Cho, Olivier Brock, Su Jeong Lim, Sung Min Youn, Younjung Park, Sangsoo Kim, Soo-Kyung Lee, Alessio Delogu, Jae W. Lee

**Affiliations:** 1 Neuroscience Section, Papé Family Pediatric Research Institute, Oregon Health and Science University, Portland, OR 97239 USA; 2 Department of Basic and Clinical Neuroscience, Institute of Psychiatry, Psychology and Neuroscience, King’s College London, London, SE5 9RS UK; 3 Department of Bioinformatics and Life Science, Soongsil University, Seoul, Korea; 4 Department of Pediatrics, Vollum Institute, Oregon Health and Science University, Portland, OR 97239 USA; 5 Present Address: Department of Biological Sciences, University at Buffalo, Buffalo, NY, 14260 USA

**Keywords:** Developmental biology, Neuroscience

## Abstract

Despite the crucial physiological processes governed by neurons in the hypothalamic arcuate nucleus (ARC), such as growth, reproduction and energy homeostasis, the developmental pathways and regulators for ARC neurons remain understudied. Our single cell RNA-seq analyses of mouse embryonic ARC revealed many cell type-specific markers for developing ARC neurons. These markers include transcription factors whose expression is enriched in specific neuronal types and often depleted in other closely-related neuronal types, raising the possibility that these transcription factors play important roles in the fate commitment or differentiation of specific ARC neuronal types. We validated this idea with the two transcription factors, Foxp2 enriched for Ghrh-neurons and Sox14 enriched for Kisspeptin-neurons, using Foxp2- and Sox14-deficient mouse models. Taken together, our single cell transcriptome analyses for the developing ARC uncovered a panel of transcription factors that are likely to form a gene regulatory network to orchestrate fate specification and differentiation of ARC neurons.

## Abstract (web summary)

Despite the crucial physiological processes governed by neurons in the hypothalamic arcuate nucleus (ARC), the developmental pathways and regulators for ARC neurons remain understudied. In this study the authors use single cell RNA-seq analyses of mouse embryonic ARC to identify cell type-specific markers for developing ARC neurons and give key insight into the underlying developmental pathways and regulators.

## Introduction

The hypothalamus, consisting of multiple nuclei, centrally regulate diverse homeostatic processes in the body. In particular, the hypothalamic arcuate nucleus (ARC) directly responds to peripheral cues due to its anatomic location in proximity to the blood stream. This allows the ARC to serve as a primary gatekeeper and processor for the peripheral signals in directing the growth, energy balance, reproduction and other behaviors in response to such cues^1,2^. Decades of studies significantly enhanced our understanding of the physiological role of the ARC neurons^1,2^. The highly interconnected actions among the ARC neurons in orchestrating the body homeostasis raise the intriguing possibility that the distinct ARC neuronal types are generated in a coordinated manner during development. However, the gene regulatory network that governs the production of ARC neurons remains poorly understood.

The ARC consists of many types of neurons expressing specific sets of neuropeptides that elicit disparate physiological actions. Agrp-neurons release the neuropeptides NPY and AgRP, which enhance food intake and reduce energy expenditure, whereas Pomc-neurons play the opposite roles by secreting the neuropeptides αMSH (cleaved from POMC) and CART^3^. Ghrh-neurons release the neuropeptide GHRH, which triggers secretion of the growth hormone (GH) from the pituitary gland^2^. GH, in turn, induces the hepatic expression of insulin-like growth factor 1 (IGF1) that controls bone epiphyses, muscle and adipose tissue development, growth plates development, and glucose homeostasis^4^. Kisspeptin-neurons (aka Kiss1-neurons) control reproduction by releasing the *Kiss1*-encoded neuropeptide Kisspeptin, which triggers the secretion of gonadotrophin-releasing hormone (GnRH) from the hypothalamic Gnrh-neurons^5^. There appear to be several distinct types of tyrosine hydroxylase (Th)^+^ neurons in the ARC. Th^+^ tuberoinfundibular dopamine (TIDA)-neurons control prolactin secretion from the pituitary^6^. Another type of Th^+^ neurons has been proposed to enhance feeding by activating Agrp-neurons and simultaneously blocking Pomc-neurons^7^. At least a subset of Ghrh-neurons was reported to express Th^8^.

Using GFP reporter mice, cell type-specific transcriptomes of adult Agrp-neurons and Pomc-neurons have been determined^9^. Recent advances in single cell transcriptomics analyses provided a new tool to catalog cell types in an unprecedented manner^10^. Using a single cell RNA-sequencing (scRNA-seq) analysis, neuronal composition of the adult hypothalamus has been analyzed^11–14^. Further, the recent scRNA-seq analysis of the adult ARC revealed that ARC neuronal composition is more complex than previously recognized^15^. This study identified 24 distinct ARC neuronal types^15^, including three types of Pomc-neurons, two types of Agrp-neurons (named Sst-expressing Agrp^Sst^-neurons, and Gm8773-expressing Agrp^Gm8773^-neurons), and six types of Th^+^ neurons (Ghrh-neurons and five additional types of Th^+^ neurons).

Despite the progress in understanding the cellular configuration of adult ARC, however, the developmental mechanisms by which these ARC neuronal types gain their unique cellular identities and the developmental relationships among distinct ARC neuronal types whose physiological actions are closely interconnected remain unclear. To address these important issues, we performed scRNA-seq analysis of the developing ARC and identified cell type-specific transcription factors and other markers for ARC neurons, which control growth, metabolism and reproduction, at the stage when they begin to emerge from the progenitor zone and acquire their distinct neuronal identities. To test if the transcription factors that are newly identified as markers of specific types of ARC neurons in the embryonic hypothalamus are important for ARC development, we further investigated and validated the vital role of the two transcription factors Foxp2, specifically enriched in Ghrh-neurons, and Sox14, enriched in Kisspeptin-neurons. Our results revealed that Foxp2 and Sox14 are important for the production of Ghrh-neurons and Kisspeptin-neurons and the control of growth and reproduction, respectively. Overall, our study identified comprehensive transcriptome profiles for key ARC neurons under development and provided a list of strong candidate transcription factors that are likely to act as key determinants for the fate and differentiation trajectory for these ARC neurons.

## Results

### scRNA-seq analysis of the developing ARC

**Fig. 1:**
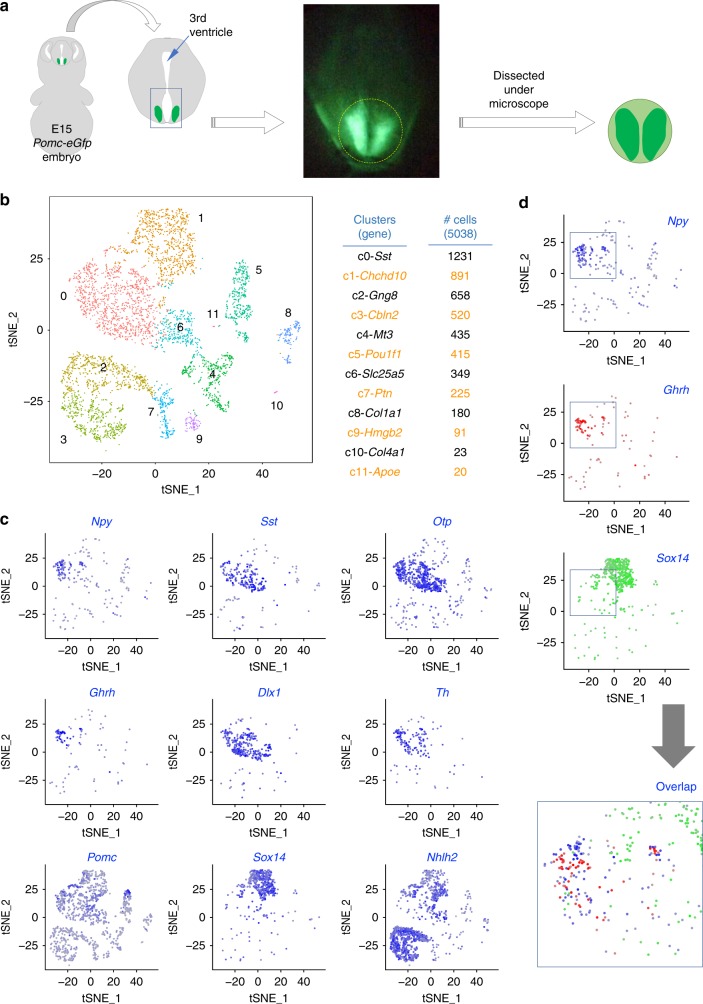
scRNA analysis of E15 ARC. **a** Schematics for dissecting out the developing ARC region from E15 *Pomc-eGfp* embryos. **b** Spectral tSNE plot of 5038 cells from E15 ARC, colored according to gene expression, reveal 12 clusters of cells (the clusters c0 to c11). The number of cells (#) in each cluster and the most specific gene in each cluster are as shown. **c** Spectral tSNE plot of 5038 cells from E15 ARC, colored for the expression of representative key marker genes of ARC neurons, *Npy/Sst/Otp* for Agrp^Sst^-neurons, *Ghrh/Dlx1* for Ghrh-neurons, *Dlx1/Th* for Th-neurons, *Pomc/Nhlh2* for POMC-neurons, and *Sox14/Nhlh2* for Kisspeptin-neurons. **d** Overlap of three spectral tSNE plots of 5038 cells from E15 ARC, colored differently for *Npy* (blue), *Ghrh* (red) and *Sox14* (green), reveals that their expressions in the cluster c0 are mutually exclusive, suggesting that the cluster c0 likely represents a pool of multiple ARC neuronal types

In the pursuit of a better understanding of the developmental principles for ARC neurons, it is imperative to capture the gene expression profiles of ARC neurons during the developmental stage when their fates are being specified because developmental regulators are often transiently expressed during cell fate specification or differentiation but are not maintained in neurons of the mature brain^16^. In developing ARC, *Pomc* begins to be expressed at E10.5 and marks a subset of ARC neuronal progenitors, which gives rise to not only Pomc-neurons but also Kisspeptin-neurons, a subset of Agrp-neurons and Ghrh-neurons^17–19^. Notably, *Npy*, *Ghrh*, and *Kiss1* (fate marker genes of Agrp-neurons, Ghrh-neurons, and Kisspeptin-neurons) mRNAs begin to be expressed in the ARC at E13.5, E14.5, and E13.5, respectively, although a full complement of neuropeptides for each ARC neuronal type emerges later^16^. For instance, while presumptive Agrp-neurons express Npy and Otp, the transcription factor critical for Agrp-neuronal fate^17^, at E15, Agrp expression is not detected before E18.5 to P0^16,20^. Likewise, the induction of Kiss1 protein begins to be detected only beyond E17.5^16^. Therefore, for our scRNA-seq analysis, we chose E15 as the developmental stage to observe multiple ARC neuronal types that are still undergoing development. Notably, *Pomc-eGfp* transgenic mice^21^, in which the ARC is clearly demarcated with the expression of GFP, enabled us to dissect out only the ARC from the embryonic brains (Fig. 1a). The cells from the pooled ARCs were then dissociated and subjected to scRNA-seq analyses^10^. The unsupervised clustering of 5038 cells using Seurat^22^ identified 12 clusters c0 to c11 (Fig. 1b; Supplementary Data 1). Our cellular identity data reveals that over 98% of cells in the clusters c0-c3 (containing ~65.5% of cells that we analyzed), as well as 24–78% of cells in the clusters c4–c10 (containing ~34% of cells that we analyzed) express the neuronal marker *Tubb3* (Supplementary Fig. 1). Also, 31–65% of cells in the clusters c0, c1, c4, c6, and c9 express the ARC progenitor marker gene *Nkx2-1* (Supplementary Fig. 1). Only the cluster 11 (containing ~0.4% of cells that we analyzed) was clearly identifiable to contain non-neuronal *Aif1*^+^ macrophages (Supplementary Fig. 1). Also, unlike the scRNA-seq results for adult ARC neurons^15^, most of our E15 scRNA-seq clusters do not express markers of differentiated non-neuronal cells such as ependymal cells, oligodendrocytes and other cell types, consistent with the notion that these non-neuronal cell types emerge at later developmental stages. Therefore, our cellular identity dataset (Supplementary Fig. 1) suggests that most cells in our E15 scRNA-seq analysis represent developing neurons and neural progenitors and very little non-neuronal cells.

To systematically investigate the cellular identity of the clusters c0 to c11, we compared their transcriptome profiles to those of the 24 adult ARC neuronal types^15^. We made a list of the genes specifically and significantly enriched in each cluster (positive value for log2 fold changes, *FDR* < 0.05), and then deduced percentage of these genes that belong to the top 200 most specific genes in each adult ARC neuronal type (Supplementary Fig. 2). These analyses suggested that many clusters have the potential relationship to multiple adult ARC neuronal types (Supplementary Fig. 2). In particular, closer examination of the transcriptomes of the clusters c0 to c11 (Supplementary Data 1) revealed that the cluster 0 expressed markers of multiple ARC neuronal types, including *Npy*, *Sst*, and *Otp*^17^ (for Agrp-neurons), *Ghrh* (for Ghrh-neurons), *Dlx1*^17^ and *Th* (for Th^+^ neurons including Ghrh-neurons), *Pomc* (for Pomc-neurons), and *Nhlh2* (for Pomc-neurons and Kisspeptin-neurons)^23,24^ (Fig. 1c). These results raise the possibility that the cluster 0 represents a pool of multiple types of developing ARC neurons, which share transcriptome profiles to be identified as a single cluster among ARC cells at this developmental stage.

### Transcriptomes of developing neurons in the ARC

**Fig. 2:**
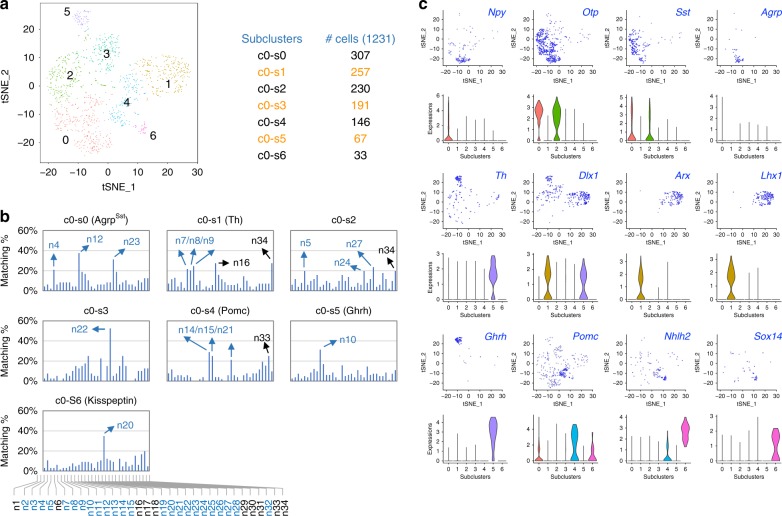
Reclustering of the cluster c0. **a** Spectral tSNE plot of 1,231 cells from the cluster c0, colored according to gene expression, reveal 7 subclusters of cells (the subclusters c0-s0 to c0-s6). The number of cells (#) in each subcluster is as shown. **b** Similarity of the E15 subclusters c0-s0 to c0-s6 to the adult neurons n1 to n34, which include 24 ARC neurons highlighted in blue, was determined by calculating what % of genes specifically enriched in each subcluster belong to the top 200 genes enriched in each of the adult neurons n1 to n34. All seven subclusters showed significant similarity to at least one type of the adult neurons. **c** Spectral tSNE plot of 1,231 cells in the cluster c0, colored for the expression of representative key markers of ARC neurons, *Npy/Otp/Sst/Agrp* for Agrp^Sst^-neurons, *Th/Dlx1/Arx/Lhx1* for Th-neurons, *Th/Dlx1/Ghrh* for Ghrh-neurons, *Pomc/Nhlh2* for Pomc-neurons, and *Nhlh2/Sox14* for Kisspeptin-neurons. Violin plots for each gene are also shown

In support of the idea that the cluster 0 is composed of multiple ARC neuronal types that are taking differentiation steps, the cells in the cluster 0 expressed markers of Agrp-neurons, Ghrh-neurons, Th-neurons, Pomc-neurons, and Kisspeptin-neurons in a relatively exclusively manner (Fig. 1c, d). To probe the heterogeneity of the cluster 0, we performed an unsupervised subclustering of the cells in the cluster c0 using Seurat^22^ and identified 7 subclusters c0-s0 to c0-s6 (Fig. 2a; Supplementary Data 2). To further define the identity of cells in each subcluster, we determined what percentage of the subcluster-specific genes (positive value for log2 fold changes, *FDR* < 0.05) overlap with the top 200 most specific genes in each adult ARC neuronal type (Fig. 2b). These analyses revealed that all seven subclusters had over 20% matching score to at least one adult ARC neuronal type.

The transcriptome of the subcluster c0-s0 highly resembled that of the three adult ARC neuronal types, n4 Sst^Nts^-neurons, n12 Agrp^Sst^-neurons and n23 Sst^Unc13c^-neurons (Fig. 2b, c, Supplementary Data 2). All of these three ARC neurons express *Agrp*, and both n4 Sst^Nts^-neurons and n23 Sst^Unc13c^-neurons also express the other key Agrp-neuronal markers *Npy*, *Sst*, and *Otp*^15^, indicating a close relationship among these three Agrp^+^ neuronal types. To test if the subcluster c0-s0 represents a pool of these three Agrp^+^ neuronal types, we subjected the subcluster c0-s0 to yet another round of an unsupervised subclustering using Seurat^22^ and identified 4 subclusters c0-s0-s0 to c0-s0-s3 (Supplementary Fig. 3). Importantly, markers of the three Agrp^+^ neuronal types in the subcluster c0–s0 were still co-expressed in the subcluster c0-s0-s0 (Supplementary Fig. 3), suggesting that the cells in the c0–s0 at E15 may represent the common precursors to the three Agrp^+^ neuronal types n4 Sst^Nts^-, n12 Agrp^Sst^-, and n23 Sst^Unc13c^ in adult ARC. Of note, none of the clusters or their subclusters in our dataset showed a high similarity to another adult Agrp-neuronal type n13 Agrp^Gm8773^-neurons, but the six subclusters c2-s0, c4-s0, c5-s1, c6-s1, c7-s0, and c8-s0 show a limited similarity to these neurons (Supplementary Fig. 4–9). Some cells in these subclusters in embryonic ARC may develop to become n13 Agrp^Gm8773^-neuronal type at a later stage.

The subcluster c0-s1 exhibited the matching scores over 20% to the three types of adult ARC neurons, n7 Arx^Nr5a2^-, n8 Th^Nfib^-neurons and n9 Th^Slc6a3^-neurons (Fig. 2b, c). In the adult ARC, n8 Th^Nfib^-neurons, and n9 Th^Slc6a3^-neurons represent the two out of six *Th*^+^ neuronal types, and n7 Arx^Nr5a2^-neuron share many molecular markers with n8 Th^Nfib^-neurons and n9 Th^Slc6a3^-neurons, although they do not express a high level of *Th*^15^. Given the potential presence of other non-ARC neuronal types in the subcluster c0-s1 (Fig. 2b), we further reclustered the subcluster c0-s1 using Seurat^22^ and identified 3 subclusters c0-s1-s0, c0-s1-s1, and c0-s1-s2, and found that the subcluster c0-s1-s2 co-expresses markers of n7 Arx^Nr5a2^-, n8 Th^Nfib^-neurons and n9 Th^Slc6a3^-neurons (Supplementary Fig. 10). Our data raise the possibility that the cells in c0-s1-s2 may eventually segregate to n7 Arx^Nr5a2^-neuronal, n8 Th^Nfib^-neuronal, and n9 Th^Slc6a3^-neuronal types in adult ARC.

The high matching scores suggested that the subcluster c0-s4 represents a pool of cells that differentiate to n14^Ttr^, n15^Anxa2^ and n21^Glipr1^ Pomc-neurons in adult ARC (Fig. 2b, c). Since this subcluster appears to contain at least one non-ARC neuronal type (Fig. 2b), it was further subclustered using Seurat^22^ to the subclusters c0-s4-s0 and c0s4-s1 (Supplementary Fig. 11). Interestingly, the subcluster c0-s4-s1 was similar to n14^Ttr^ Pomc-neurons in the adult ARC (Supplementary Fig. 11). Our subclusterings also revealed that the subcluster c1-s3 shows a limited similarity to Pomc-neurons (Supplementary Fig. 12), raising the possibility that some cells in this subcluster in embryonic ARC may develop to become Pomc-neuronal type(s) at a later stage. In addition, the subcluster c5-s2 was similar to n15 Pomc^Anxa2^-neurons in the adult ARC (Supplementary Fig. 6). Our data suggest that the cells in c5-s2 may develop to become n15 Pomc^Anxa2^-neuronal type in adult ARC.

The transcriptome profiles of subclusters c0-s5 and c0-s6 at E15 decisively resembled those of Ghrh-neurons and Kisspeptin-neurons in adults, respectively (Fig. 2b, c, Supplementary Data 2). Our analyses also indicated the relationship between the embryonic subclusters c0-s2 and c0-s3 and the adult n27 Tbx19-neurons and n22 Tmem215-neurons^15^, respectively (Supplementary Fig. 13, Fig. 2b). The physiological roles of n27 Tbx19-neurons and n22 Tmem215-neurons remain to be determined.

**Fig. 3:**
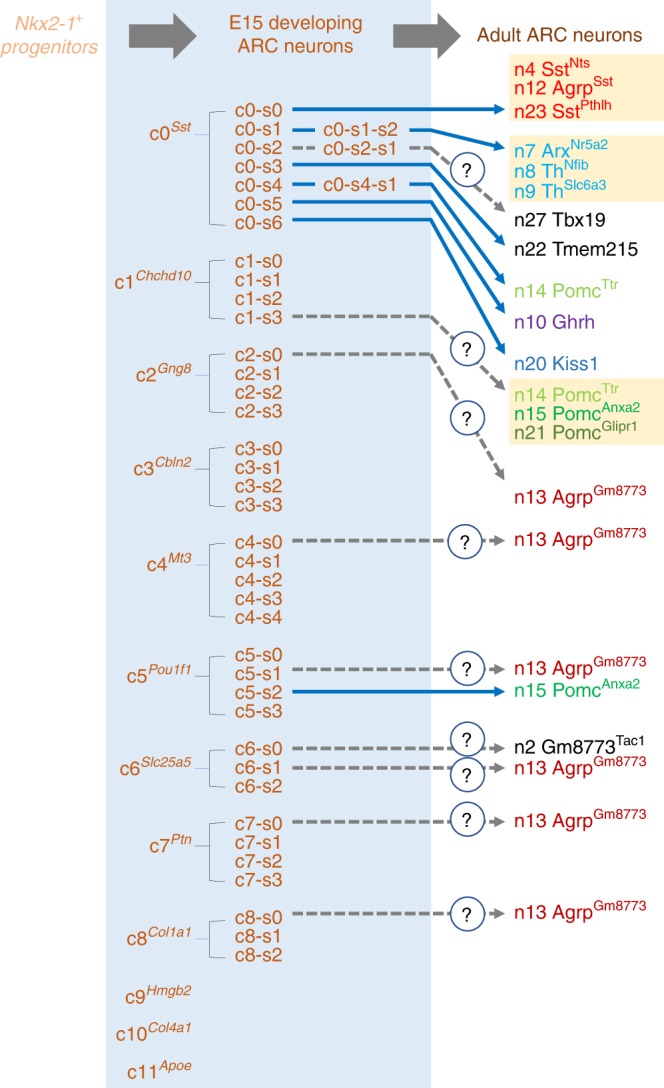
Developing ARC neurons from our scRNA-seq analysis of E15 ARC. Progenitors labeled by the transcription factor gene *Nkx2-1* eventually give rise to different neuronal types in the adult ARC. Relative to the 24 ARC neuronal types defined by a recent RNA-seq analysis of adult ARC^15^, our scRNA-seq analysis of E15 ARC reveals several developing ARC neuronal types. Interestingly, cells in the subclusters c0-s0, c0-s1-s2, and c1-s3 appear to serve as common progenitors to multiple neuronal types in the adult ARC. Cells in the subcluster c0-s0 may represent developing neurons that later segregate to n4 Sst^Nts^-neurons, n12 Agrp^Sst^-neurons, and n23 Sst^Pthlh^-neurons. Cells in the subcluster c0-s1-s2 may become n7 Arx^Nr5a2^-neurons, n8 Th^Nfib^-neurons, and n9 Th^Slc6a3^-neurons, while cells in the subcluster c1-s3 may segregate to n14 Pomc^Ttr^-neurons, n15 Pomc^Anxa2^-neurons n21 Pomc^Glipr1^-neurons. Cells in the subclusters c0-s3, c0-s4-s1, c0-s5, and c0-s6 may also give rise to n22 Tmem215-neurons, n14 Pomc^Ttr^-neurons, n10 Ghrh-neurons, and n20 Kisspeptin-neurons, respectively

Together, our unsupervised clustering analyses (Fig. 2b, Supplementary Fig. 2–13), combined with the expression pattern of the known fate markers (Fig. 2c, Supplementary Data 2, Supplementary Data 3), identified the gene expression profiles of the embryonic neuronal groups that are likely to differentiate to Agrp^Sst^-neurons, n8^Nfib^/n9^Slc6a3^ Th-neurons, n22 Tmem215-neurons, Pomc-neurons, Ghrh-neurons, and Kisspeptin-neurons in the mature ARC (Fig. 3).

### Identification of new markers for developing ARC neurons

A limited number of markers available for assessing the developing ARC neuronal types remains a major challenge in studying the gene regulatory network governing production of diverse ARC neuronal types. Our scRNA-seq database for the subclusters c0-s0, c0-s1-s2, c0-s3, c0-s4-s1, c0-s5, c0-s6, and others (Fig. 3, Supplementary Data 2, 3) provide new markers required for systematically defining the developmental trajectory of ARC neurons in wild-type and mutant mouse models, as well as an invaluable and much needed toolkit to identify the transcriptional regulators, which play important roles in the ARC development (Supplementary Data 2, 3).

**Fig. 4:**
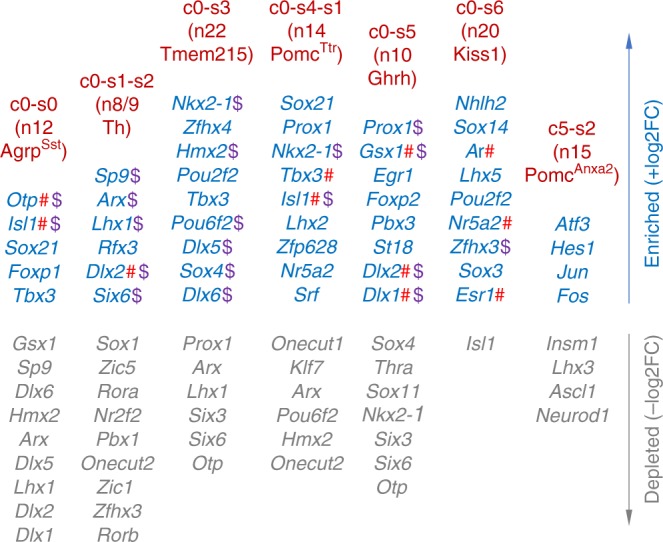
Transcription factors enriched in E15 developing ARC neurons. We listed transcription factor transcripts in the subclusters c0-s0, c0-s1-s2, c0-s3, c0-s4-s1, c0-s5, c0-s6, and c5-s2, which show relatively stronger similarity to adult ARC neurons. Transcripts for the transcription factors that have been already implicated in the development of ARC neurons are denoted by #, while transcripts for the transcription factors showing the unique expression pattern of being enriched in one or more subclusters while being excluded from other subclusters are highlighted as $

To identify the key transcriptional regulators for the fate determination and differentiation of ARC neurons, we analyzed the differential transcriptome profiles that divided the cells in the cluster 0 into 7 subclusters (Fig. 2a). The cells in the cluster 0 shared the overall gene expression profile to be identified as a single cluster when a total pool of E15 ARC cells was analyzed, but the distinguishing features among the cluster 0 cells were strong and consistent enough to divide them into 7 subcluster groups, whose properties resemble the traits of adult ARC neuronal types. Thus, we reasoned that the transcription factors that are strongly enriched in a specific subcluster while being depleted in other subclusters are strong transcriptional regulator candidates that determine the identity of each neuronal type and control the ensuing developmental steps. Our bioinformatics analyses identified the transcripts for 38 transcription factors that are specifically enriched in the subclusters c0-s0, c0-s1-s2, c0-s3, c0-s4-s1, c0-s5, and c0-s6 (Fig. 4). Supporting our rationale for this screen, the list included transcripts for the nine transcription factors that have been reported to regulate the development of specific ARC neuronal types (indicated with #, Fig. 4); *Isl1* and *Otp* for Agrp-neurons^17,20^, *Dlx2* for Th-neurons^25^, *Gsx1* and *Dlx1/2* for Ghrh-neurons^17,26^, *Isl1* and *Tbx3* for Pomc-neurons^20,27,28^ and *Nr5a2* and the two sex hormone receptors *Ar/Esr1* for Kisspeptin-neurons^29,30^. In addition, many of these transcription factor transcripts are highly enriched in one cell type while their expressions are depleted from other cell types (indicated with $, Fig. 4). For instance, *Otp* is enriched in c0-s0, related to n12-Agrp^Sst^, and depleted in other subclusters c0-s3 and c0-s5, related to n22-Tmem215 and n10-Ghrh neurons, respectively (Fig. 4), leading us to predict that Otp promotes Agrp-neuronal fate and possibly represses Tmem215-neuronal and Ghrh-neuronal identity. This prediction is consistent with our previous finding that Otp is required for the production of Agrp-neurons and the aberrant induction of Otp in prospective Ghrh-neurons switch the cells to Agrp-neuronal types^17^. In addition, we found transcripts for the four transcription factors that are specifically enriched in the subcluster c5-s2 (Fig. 4). Notably, the cluster c5 is rather distant from the cluster c0 in the spectral tSNE plot (Fig. 1b), suggesting that the cells in the clusters c5 and c0 may take separate developmental lineages. Consistent with this prediction, the four transcription factor transcripts in the subcluster c5-s2 were not depleted from the c0-derived subclusters c0-s0, c0-s1-s2, c0-s3, c0-s4-s1, c0-s5, and c0-s6.

**Fig. 5:**
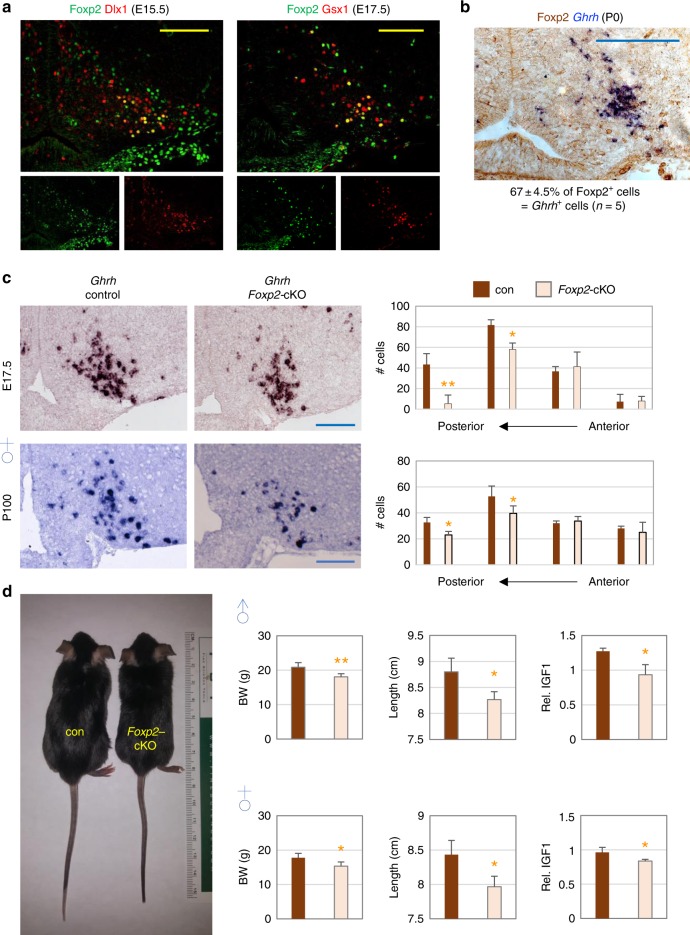
Crucial roles of Foxp2 in the development of Ghrh-neurons. **a** IHC with antibodies against Foxp2 and either Dlx1 or Gsx1 reveals co-expression of Foxp2 and Dlx1/Gsx1 in a subset of E15.5/E17.5 ARC neurons. **b** ISH for *Ghrh* (blue) combined with IHC against Foxp2 (brown) reveals co-expression of Foxp2 with *Ghrh* in P0 ARC. **c** ISH against *Ghrh* reveals that the number of *Ghrh*^+^ cells (i.e., Ghrh-neurons) in the ARC is significantly reduced in *Foxp2*-cKO mice relative to control mice at both E17.5 and P100 (in both sexes). The number of mice per each genotype was 3–5. The distance between serial sections was 132 μm, and the images shown are for the second posterior sections. **d** Dwarfism of P50 *Foxp2*-cKO male and female mice, accompanied by significant reductions in body weight (BW), linear growth (length, cm), and the relative expression levels of IGF1 in the liver. The number of mice for each genotype was 3–5. All scale bars are for 100 μm. Statistical differences were determined by Student’s *t*-test (**c**, **d**); *p* < 0.05 (one asterisk) and *p* < 0.01 (two asterisks)

**Fig. 6:**
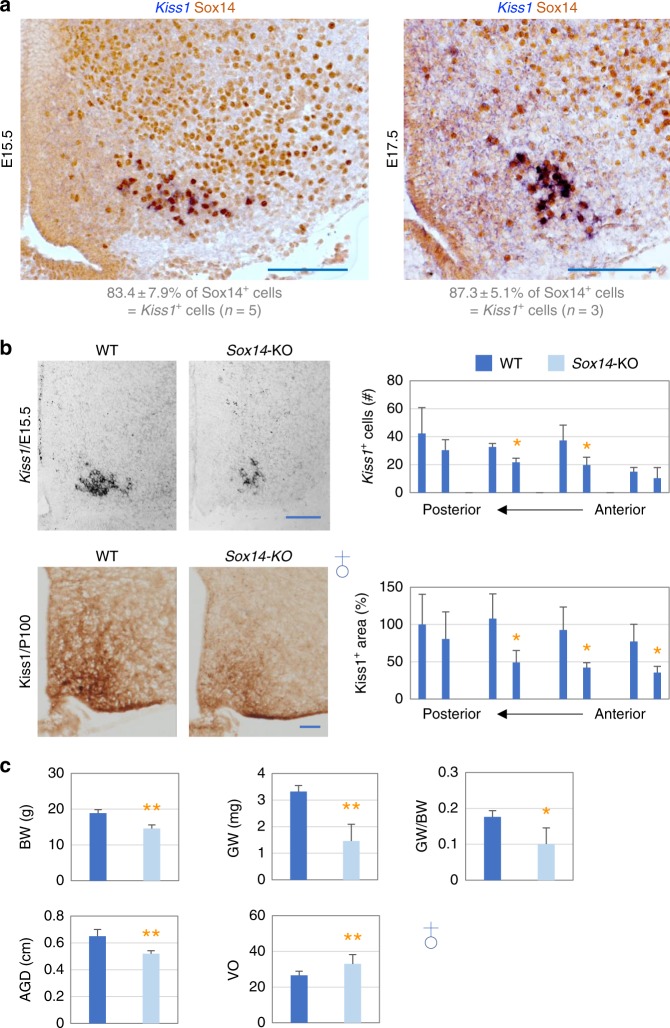
Critical roles of Sox14 in the development of Kisspeptin-neurons. **a** ISH for *Kiss1* (blue) combined with IHC against Sox14 (brown) reveal co-expression of Sox14 with *Kiss1* in E15.5 and E17.5 ARC. **b** Significantly reduced expression of *Kiss1* mRNA in the ARC of E15.5 embryos (ISH) and Kiss1 protein in the ARC of P100 female control and *Sox14*-KO mice (IHC). Serial anterior-posterior sections are apart from each other by ~120 μm, and the images shown are for the second anterior sections. **c** Body weight measurement, as well as assessment of reproduction phenotypes, GW (for gonad weight), AGD (for anogenital distance) and VO (for vaginal opening), for P100 female mice. The number of mice per each genotype was 3–15. All scale bars are for 100 μm. Statistical differences were determined by Student’s *t*-test (**b**, **c**); *p* < 0.05 (one asterisk) and *p* < 0.01 (two asterisks)

In addition, we confirmed cell type-specific expression pattern of many markers that our scRNA-seq identified using in situ hybridization (ISH), combined with immunohistochemistry (IHC), and dual/triple IHC analyses (e.g., Supplementray Fig. 14, Supplementray Fig. 15, Fig. 5a, b, Fig. 6a, Supplementray Fig. 16c), verifying the quality of our datasets.

Together, our unbiased single cell transcriptome analyses revealed a list of cell type-specific markers, including 42 transcription factors that are likely to play crucial roles in the fate determination and differentiation of key ARC neuronal types in the developing hypothalamus.

### Foxp2 is critical for Ghrh-neuronal development

To further validate if our newly identified transcription factors in developing ARC neurons are indeed important for ARC neuronal development, we investigated the role of Foxp2 that our bioinformatics analyses determined to be highly enriched in the subcluster c0-s5, representing developing Ghrh-neurons (Fig. 4). Consistent with our scRNA-seq analyses, the dual IHC assays revealed that Foxp2 is co-expressed with Dlx1, which marks both Th-neurons and Ghrh-neurons^17^, in the lateral zone of the ARC where Ghrh neurons reside at E15.5 (Fig. 5a). Similar co-expression of Foxp2 and Gsx1, the Ghrh-neuronal marker^26^, was also observed at E17.5 (Fig. 5a). Moreover, Foxp2 is expressed in Ghrh-neurons at P0 (Fig. 5b), indicating that Foxp2 expression is maintained specifically in Ghrh-neurons at P0. Next, by crossing *Foxp2*^*f/f*^ mice^31^ to *Nkx2-1-Cre* mice^32^, we created conditional knockout (cKO) mice in which *Foxp2* is deleted in developing hypothalamus. *Foxp2*-cKO mice showed a significantly lower number of *Ghrh*^+^ cells than control mice at both E17.5 and P100 (Fig. 5c). In contrast, *Npy*^+^ cells in *Foxp2*-cKO mice did not reduce relative to control mice (Supplementary Fig. 16a). These results suggest that Foxp2 is critical for the development of Ghrh-neurons but not Agrp-neurons. Consistent with the reduced number of Ghrh-neurons, *Foxp2*-cKO mice showed deficits in GH signaling, including significant decreases in body weight, linear length (height) and the hepatic IGF1 levels (Fig. 5d). Taken together, these results strongly support critical roles of Foxp2 in the development of Ghrh-neurons and validate our finding of Foxp2 in the transcriptome of developing Ghrh-neurons (Fig. 4).

### Sox14 is crucial for Kisspeptin-neuronal development

Given our finding that *Sox14* transcript is specifically expressed in developing Kisspeptin-neurons (Fig. 4), we examined if Sox14 is important for the development of Kisspeptin-neurons. Our dual IHC assays using the previously reported Sox14 antibody^33^ (also see Supplementary Fig. 16b) found that Sox14 is co-expressed with the Kisspeptin-neuronal marker Esr1^30^ in a subset of cells at E15.5 (Supplementary Fig. 16c). Further validating that this subset of cells represents Kisspeptin-neurons, dual IHC assays using antibodies against Sox14 and Kiss1 (Kiss1 proteins begin to be detected as early as E17.5) revealed that Sox14 and Kiss1 are co-expressed in a similar subset of cells at E17.5 (Supplementary Fig. 16c). Similar co-expression of Sox14 and Kiss1 was also confirmed in our IHC assays (for Sox14) in combination with ISH (for *Kiss1*) at both E15.5 and E17.5 (Fig. 6a). Consistent with this expression pattern, *Sox14*-KO mice showed a markedly lower level of *Kiss1* mRNA and Kiss1 protein at both E15.5 and P100, respectively (Fig. 6b). Moreover, *Sox14*-KO mice showed a significant decrease in gonad weight, as well as anogenital distance (AGD) in both males and females (Fig. 6c, Supplementary Fig. 16d). Unexpectedly, both male and female *Sox14*-KO mice also showed a significant reduction in body weight (Fig. 6c, Supplementary Fig. 16d). Even when normalized to body weight, gonadal weight was still significantly reduced (Fig. 6c, Supplementary Fig. 16d). Moreover, the vaginal opening was clearly delayed in female *Sox14*-KO mice (Fig. 6c). Consistent with these results, *Sox14*-KO mice were completely sterile despite our extensive breeding efforts. Taken together, our results suggest that Sox14 is critical for Kisspeptin-neuronal development, which likely underlies the infertility of *Sox14*-KO mice.

## Discussion

In comparison to the reported scRNA-seq analysis of the adult ARC neurons^15^, our scRNA-seq analysis of developing ARC neurons at E15 provides several important insights into the development of ARC neurons (Fig. 3). First of all, while the developing ARC at E15 contains the seven subclusters that are nicely related to the adult counterpart ARC neurons^15^ (c0-s0, c0-s1-s2, c0-s3, c0-s4-s1, c0-s5, c0-s6, and c5-s2, Fig. 3), the majority of cells at this stage are distantly related to the adult ARC neurons. Together with our cellular identity analysis indicating that most cells represent developing neurons and progenitors (Supplementary Fig. 1), these results suggest that cells in the developing ARC at E15 are mostly at an early stage of development before manifesting clear adult neuronal cell type identities. Secondly, it is noted that the cluster c0 from our first unsupervised clustering represents a pool of a few neuronal types, including Agrp^Sst^-neurons and Kisspeptin-neurons, as well as a subset of Pomc^+^ and Th^+^ neurons (including Ghrh-neurons) (Fig. 3). These results demonstrate that these cell types are closely related to each other in developmental lineages, consistent with the previous results showing that *Pomc*^+^ progenitor cells produce not only Pomc-neurons but also Kisspeptin-neurons and a subpopulation of Agrp-neurons^18,19^. Our recent report also demonstrated an unexpected plasticity between Agrp-neuronal and Ghrh-/Th-neuronal fates^17^, and these results are validated by the co-clustering of developing Agrp-neurons and Ghrh-/Th-neurons in the cluster c0 (Fig. 3). Thirdly, some subclusters appear to be a pool of multiple neuronal types (Fig. 3). Overall, our results suggest that the development of many ARC neuronal types and segregation of the embryonic hybrid subclusters (e.g., c0-s0, c0-s1-s2, and c1-s3, Fig. 3) to individual cell types occur at later developmental stages. Based on these observations, we believe that additional scRNA-seq analyses of developing ARC neurons at stages later than E15 are needed for a more comprehensive understanding of the developmental processes of ARC neurons. Finally, it is interesting to note that the clusters c0 and c5 are rather separated from each other in the tSNE plot (Fig. 1b). The notion that the cells in the subclusters c0-s4-s1 and c5-s2 may develop to become n14 Pomc^Ttr^-neurons and n15 Pomc^Anxa2^-neurons in adult ARC, respectively, combined with our finding that the cells in the cluster c0 yield Agrp^Sst^-neurons, n8/n9 Th-neurons, n22 Tmem215, n10 Ghrh-neurons, and n20 Kisspeptin-neurons (Fig. 3), raises an interesting possibility that *Pomc*^+^ progenitors giving rise to n14 Pomc^Ttr^-neurons (i.e., c0-s4-s1), but not n15 Pomc^Anxa2^-neurons (i.e., c5-s2), may eventually develop to become Agrp^Sst^-neurons, n8/n9 Th-neurons, n22 Tmem215, n10 Ghrh-neurons, and n20 Kisspeptin-neurons in adult ARC. These results also suggest that n15 Pomc^Anxa2^-neurons may be quite distinct from n14 Pomc^Ttr^-neurons in developmental lineages.

Our finding of 38 transcription factors whose transcripts are specifically enriched in the subclusters c0-s0, c0-s1-s2, c0-s3, c0-s4-s1, c0-s5, and c0-s6, as well as 4 transcription factor transcripts in the subcluster c5-s2 (Fig. 4) is believed to be a major contribution to our efforts to understand the developmental processes of ARC neurons, as we reason that they likely play critical roles in the development of specific ARC neuronal types. Firstly, we were able to further segregate cells in the cluster c0 (which share the overall gene expression profile to be collectively identified as a single cluster) to 7 different subcluster groups, suggesting that transcription factors enriched in each subcluster may represent the distinguishing features among the cluster c0 cells that determine the identity of neuronal type for each subcluster and control the ensuing developmental steps. Secondly and consistent with this idea, the list included nine transcription factors that have been already reported to regulate the development of specific ARC neuronal types (indicated with #, Fig. 4)^17,20,25–30^. These include the recent finding for the transcription factor Tbx3 in the development of Pomc-neurons^28^. Notably, Tbx3 is highly enriched in both developing Agrp^Sst^-neurons and Pomc^Ttr^-neurons in our E15 scRNAseq dataset (Fig. 4), and therefore Tbx3 may also control the development of Agrp^Sst^-neurons. Sox21-deficine mice showed enhanced energy expenditure^34^, and it will be interesting to investigate whether this phenotype involves the specific enrichment of the transcription factor Sox21 in our scRNA-seq dataset for developing Agrp-neurons and Pomc-neurons (Fig. 4), both of which control energy expenditure. Hypothalamic Atf3, found in our developing Pomc^Anxa2^-neurons, has also been implicated in regulating glucose and energy metabolism^35^, and it will be an interesting future study to investigate whether Atf3 is involved in the development of Pomc^Anxa2^-neurons. Thirdly, many of these transcription factors are highly enriched in specific cell types while their expressions are depleted from other cell types (indicated with $ symbol, Fig. 4). Notably, many of these transcription factors in the list also show similar expression pattern in the adult ARC, being enriched in specific adult ARC neuronal types while being depleted in other adult ARC neuronal types^15^. For instance, the transcription factor Prox1, enriched in developing Ghrh-neurons (i.e., c0-s5) and Pomc^Ttr^-neurons (i.e., c0-s4-s1), is also highly enriched in adult Ghrh-and Pomc^Ttr^-neurons while it is depleted in the remaining 22 adult neuronal types in the ARC^15^. These results raise an interesting possibility that all our transcription factors may have manifested similar expression patterns in E15 ARC if our scRNA-seq experiments achieved deeper sequencing depth. Indeed, most of our transcription factors were found to be depleted in other subclusters when we adjusted the FDR to more than 0.05. Finally, in further functional validation of our list of 42 transcription factors (Fig. 4), we demonstrated that the two transcription factors Foxp2 and Sox14 are critical for the development of Ghrh-neurons and Kisspeptin-neurons, respectively (Fig. 5, Fig. 6, Supplementary Fig. 16). Notably, we made unexpected observation for body weight reduction in *Sox14*-KO mice (Fig. 6c, Supplementary Fig. 16d). This reduction in body weight can be due to the action of Sox14 in other cell types, and future experiments with Kisspeptin-neuron specific cKO model for *Sox14* will clarify this issue. Overall, our results strongly suggest that our newly discovered transcription factors are likely the key fate determinants of developing ARC neurons and therefore their further dissection will greatly advance our understanding of how ARC neurons develop during embryogenesis.

In summary, this report describes the first single cell transcriptomes of several developing ARC neuronal types. Moreover, these datasets include key fate-determining transcription factors, and further dissection of their roles in developing ARC will greatly advance our understanding of how ARC neurons develop during embryogenesis. Finally, the regulatory elements associated with our new marker genes (Supplementary Data 2, 3) can be developed to construct new transgenic mouse lines, in which the expression of Cre recombinase can be directed to individual developing ARC neuronal types. Notably, highly cell type-specific mouse Cre lines will expedite our efforts to understand the development of ARC neurons.

## Methods

### Animals

All mouse works were performed under approved protocols by the Institutional Animal Care and Use Committee of the Oregon Health and Science University and King’s College London. *Foxp2*^*f/f*^, *Nkx2.1-Cre*, *Pomc-eGfp*, *Sox14-KO* (*Sox14*^*Gfp/+*^ and *Sox14*^*Cre/+*^) mice^21,31,32,36,37^ were maintained in standard cages with free access to normal chow and water on a 12:12 light:dark cycle. Body weight and linear length were measured^17^. Phenotypic characterization of sexual maturation was performed on virgin animals from both sexes between P53 and P100. After anesthesia, mice were weighed and measured for their AGD before proceeding with transcardial perfusion fixation with 4% paraformaldehyde in PBS. Gonads were isolated and postfixed for 24 h, washed in PBS and weighed. The day of vaginal opening was established by daily visual inspection.

### scRNA-seq

Pregnant Pomc-eGfp mice were euthanized 15 days after detection of vaginal plugs. The ventral sides of the hypothalamus of the embryos (*n* = 6) were collected, and the GFP positive regions, which are indicative for the location of the ARC, were precisely dissected out under a dissection fluorescence microscope. All samples were combined and dissociated using standard Papain digestion^15^. The resulting cells were counted using an automated cell counter and subjected to the generation of a scRNA-seq library and sequencing^10^.

### Data processing and alignment

Raw sequencing cell barcodes were filtered to distinguish between valid cell barcodes from empty cell barcodes using an algorithm in Cellranger count v2.1.1., an analysis pipeline for Chromium single cell 3′ RNA-seq results^10^. Total unique molecular identifier (UMI) counts across all detected barcodes were rank-sorted, and the 99th percentile of the UMI counts among the top *n* barcodes (where *n* is provided with an expected recovered cell parameter; *expect-cells* set to 5300 in our case) were selected. The barcodes with total UMI counts larger than or equal to 10% of the 99th percentile values were classified as valid barcodes. Using STAR v2.4.0 with a default setting^38^, the filtered reads were aligned to mm10 (refdata-cellranger-mm10-1.2.0). Only confidently mapped (MAPQ = 255), non-PCR duplicates with valid barcodes and UMIs were employed to generate gene-barcode matrix. Among the 394,067,025 reads, 333,380,703 (84.60%) of reads were mapped.

### Filtering and normalization

Gene-barcode count matrix was read using a function *Read10X* in an R package Seurat v2.0 for downstream analysis^39^. We initialized the object with the raw data and kept all genes expressed in >3 cells and all cells with at least 200 detected genes. To further remove possible doublets and poor-quality cells, we filtered out outlier cells that have unique gene counts over 4500 or less than 200 (determined based on visual inspection over gene count distribution). Also cells with a high proportion of mitochondrial genes (>10%) were filtered out based on a report showing that if a cell is lysed, cytoplasmic RNA will be lost apart from the RNA enclosed in the mitochondria, which will be retained and sequenced as a quality control metric^40^. Further, we filtered out cells having a high level of hemoglobin gene expression (*Hba-a1* > 10) given it likely derived from blood cells. Out of 6465 cells, 5038 cell barcodes met quality control criteria and were utilized for downstream analysis. After removing unwanted cells from the dataset, the gene-barcode matrix was normalized using a global-scaling normalization method *LogNormalize*^22^, in which gene expression for each cell was normalized by the total expression, multiplied by a scale factor 10,000, and then log-transformed.

### Dimensionality reduction, clustering, and data visualization

For downstream analysis, we identified a set of highly variable genes in terms of expression level across the entire data set and focused on those rather than all genes. The average expression and dispersion (variance/mean) for each gene across all cells were calculated using *FindVariableGenes* function^22^ and placed into bins based on their average expression. Each *z*-score was then calculated for dispersion within each bin. To mitigate the effect of confounding factors, we built linear model to regress out the number of detected molecules per cell and mitochondrial gene expression. After scaling and centering the data along each variable gene, we identified 1664 highly variable genes. The scaled *z*-scored residuals of these models were used for dimensionality reduction and clustering. We performed principal component analysis on the scaled matrix to further reduce the dimensions of the data. To select a set of principal components (PC) for downstream clustering analysis, we used a statistical resampling procedure *jackStraw* in Seurat package^22^. It constructs a null distribution for PC scores and then estimates its *p*-value for each PC. For data visualization, using 14 PCs chosen as described above, we performed t-distributed stochastic neighbor embedding (tSNE) technique^41^ with the perplexity parameter^42^, an expected number of neighbors, set to the default value 30. Graph-based clustering approach in Seurat^22^ was used in which distances between the cells are calculated based on the identified PCs. To cluster the cells, a modularity optimization techniques for detecting communities in a network was used with an original Louvain algorithm within a function *FindClusters*^43^ to group cells together with a resolution parameter 0.6, which returns good results for around 6000 cells. Overall, we assigned 5038 cells into 12 different cell clusters. Subclustering was further performed using 11PCs. Cells in each initial cluster were extracted and reclustered in the same procedure with perplexity parameter set to 0.6. Violin plots were utilized to visualize the distribution of gene expression among clusters. Feature plots were drawn to color single cells according to their gene expression on a two-dimensional tSNE space.

### Cluster identity determination

We took the following steps to determine a match between our embryonic ARC clusters and the 24 adult ARC neuronal clusters^15^. First, we identified the top 50 or 200 genes that are specifically enriched in each of the adult ARC neuronal clusters n1 to n34 (by sorting the genes based on enrichment fold)^15^. Next, we made a list of the genes specifically and significantly enriched in each of our E15 clusters (positive value for log2 fold changes, *FDR* < 0.05), and then deduced percentage of these genes that belong to the above top 200 or the top 50 adult ARC genes. The top 50 genes represent the most specifically expressed genes in each adult neuronal type relative to other adult neuronal types, many of which are terminally differentiated neuronal markers. Relative to the top 50 genes, the top 51–200 genes are less specifically expressed in each adult neuronal type. Therefore, a cluster showing a match to both the top 200 genes and the top 50 genes of a specific adult ARC neuronal type represents a more terminally differentiated neuronal type relative to a cluster which shows a match to the top 200 genes but not the top 50 genes. The latter cluster consists of the cells that are undergoing differentiation to become the specific adult ARC neuronal type but yet to gain a battery of terminal differentiation markers.

### ISH and IHC

Brains were removed from the embryos (up to P0) and fixed in 4% PFA overnight, cryoprotected with sucrose, and frozen in OCT blocks, which were then sectioned using a microtome with a thickness of 12 µm per section. P50 or P100 mice were intraperitoneally injected with Avertin before performing standard perfusion with PBS and 4% PFA, followed by overnight fixation in 4% PFA. ISH was performed at 68 °C overnight with indicated RNA probes. After hybridization, slices where incubated in washing buffer (50% formamide, 1× SSC solution and 0.1% Tween20) for 1 h, blocked in MABT buffer + 4% BSA for 1 h and subsequently incubated with an anti-digoxigenin-AP antibody (11093274910 Roche, 1:5000) in MABT buffer + 2% BSA. Next day the color reaction was performed with NBT/BCIP after washing with MABT buffer. For subsequent co-staining of visualized RNA probes, hybridized sections were incubated with our homemade antibodies against Dlx1/Otp (1:1000)^17^ or Sox14 (1:1500)^33^. Next day, Vectastain ABC Elite kit (PK-6101, Vector labs) was used for the color reaction. In addition to our previously described ISH probes against *Ghrh*, *Npy* and *Kiss1*^17,20^, we also generated new RNA probes by converting hypothalamic RNA of P0 mice to cDNA using the primers in Table 1. PCR products were then digested with the indicated enzymes and ligated into pBluescript. Digoxigenin-labeled riboprobes probes were generated using T7 RNA polymerase followed by purification over a column.

**Table 1:** Primers used in this study

Cited1-ECORI-forward (FW)-ISH Cited1-XHOI-reverese (RV)-ISH	GGAATTCCTGGGGACTCTGAAGCGAG TATACTCGAGCAGCCAGAGGGAAAATCTGC
Pik3r1-HINDIII-FW-ISH Pik3r1-XHOI-RV-ISH	TATAAGCTTGGCGTGACATGTAGGCTCTCAG TATCTCGAGAAAGGTCCCATCAGCAGTGTC
Peg10-ECORI-FW-ISH Peg10- HINDIII-RV-ISH	ATGAATTCCAAGTGAAAAGAGGGTGGAAAC TATAAG CTTACTTCCTTTTCAAGCTGAGGTG
Calcr-HINDIII-FW-ISH Calcr-XHOI-RV-ISH	GATAAG CTTATCTCGGAGCGAGCAGC TATACTCGAGAAGTACAGGAATGGCTCCTG
Cbln4-BAMH1-FW-ISH Cbln4-HINDIII-RV-ISH	CAGGGATCCTGCTGGTGATAAAGATGTG CCCAAGCTTAGTCAGAACTTCCAAGTTTTCTAC
Gal- ECORI-FW-ISH Gal- HINDIII-RV-ISH	TAGAATTCTCCTGCACTGACCAGCCAC ATTAAGCTTGATTGGCTTGAGGAGTTGGC
Asb4-ECORI-FW-ISH Asb4-HINDIII-RV-ISH	GGAATTCGCAAAGTGCTACCCCAAAAGG TATAAGCTTGCAGGGGTGTCTCTTCATCC
Resp18-ECORI-FW-ISH Resp18-HINDIII-RV-ISH	GAGAATTCCGCTAGAGGGTGAAAAGTGAC TATAAGCTTGGCCTTTGGGATTACTTTGGTG
IGF1 FW IGF1 RV	TCATGTCGTCTTCACACCTCT TCCACAATGCCTGTCTGAGG

IHC was performed by incubating brain sections with antibodies against Kiss1 (Millipore AB9754, 1:500), Esr1 (Millipore 04–227, 1:500), Foxp2 (Abcam 16046, 1:2000), and Sox14 (home-made, 1:1500)^33^ overnight at 4 °C. Next day, slices were washed with PBST and incubated with secondary fluorescence antibodies followed by washing and counter staining with DAPI. For Kiss1 IHC, mice were transcardially perfused with 4% paraformaldehyde in PBS and the brains were postfixed at 4 °C for 2 h. Brains were equilibrated in 30% Sucrose/PBS, embedded in OCT freezing compound, cut on a cryotome at 30 µm and collected in ice-cold PBS. Endogenous peroxidase activity was quenched by 1% H_2_O_2_/PBS incubation for 30 min at room temperature, rinsed in PBS then blocked for 2 h at room temperature in 7% normal goat serum with 0.3%Triton X-100 in PBS, then incubated with anti-Kiss1 rabbit antiserum AC566 (kindly provided by INRA, France, 1:10,000) diluted in blocking solution for 72 h at 4 °C. All wash steps were carried out in PBS alone. Secondary biotinylated goat anti-rabbit antibody (1:500) was added for 2 h at room temperature. Sections were incubated with horseradish peroxidase avidin-biotin complex (Vectastain ABC Elite kit, PK-6101, Vector labs) diluted in 0.3% Triton X-100 in PBS for 1 h at room temperature. Colorimetric reaction was carried out in presence of diaminobenzidine substrate (Sigma) for 10 min. Sections were then dehydrated and mounted in non-aqueous medium.

### Quantification

For IGF1 quantification, small pieces of liver of avertin treated mice were collected and dissolved in Trizol using a tissue grinder. Total RNA was than extracted, converted into cDNA and IGF1 cDNA was quantified using qPCR with the primers listed in Table 1^17^. Quantification of ISH sections for *Ghrh*, *Npy*, and *Kiss1* was performed by counting the number of cells in comparable sections of control and mutant mice. For quantification of the Kiss1-positive area, ARC-containing sections from control and *Sox14*-KO brains were processed for Kiss1 immunodetection by the DAB method in parallel. Digital images were acquired under identical magnification, lighting and exposure. Digital images were converted to 8-bit and quantified using the “threshold” function in ImageJ. The same threshold value was applied across all samples.

### Reporting summary

Further information on research design is available in the Nature Research Reporting Summary linked to this article.

## Supplementary information

Supplementary Information

Reporting Summary

Description of Additional Supplementary Files

Supplementary Data 1

Supplementary Data 2

Supplementary Data 3

## Data Availability

scRNA-seq data that support the findings of this study have been deposited in the National Center for Biotechnology Information Gene Expression Omnibus (GEO) and are accessible through the GEO Series accession number GSE126480. All other relevant data are available from the corresponding author on request.
