# Author Correction: Single cell transcriptome analysis of developing arcuate nucleus neurons uncovers their key developmental regulators

**DOI:** 10.1038/s41467-020-14964-z

**Published:** 2020-03-10

**Authors:** Christian Huisman, Hyeyoung Cho, Olivier Brock, Su Jeong Lim, Sung Min Youn, Younjung Park, Sangsoo Kim, Soo-Kyung Lee, Alessio Delogu, Jae W. Lee

**Affiliations:** 1 Neuroscience Section, Papé Family Pediatric Research Institute, Oregon Health and Science University, Portland, OR 97239 USA; 2 Department of Basic and Clinical Neuroscience, Institute of Psychiatry, Psychology and Neuroscience, King’s College London, London, SE5 9RS UK; 3 Department of Bioinformatics and Life Science, Soongsil University, Seoul, Korea; 4 Department of Pediatrics, Vollum Institute, Oregon Health and Science University, Portland, OR 97239 USA; 5 Present Address: Department of Biological Sciences, University at Buffalo, Buffalo, NY 14260 USA

**Keywords:** Developmental biology, Neuroscience

Correction to: *Nature Communications* 10.1038/s41467-019-11667-y, published online 16 August 2019.

The original version of this Article contained an error in Fig. 4, where the transcription factors enriched in E15 developing ARC neurons were not correctly listed. The correct version of Fig. 4 is: which replaces the previous incorrect version: This has been corrected in both the PDF and HTML versions of the Article.

The original version of this Article contained multiple errors in the Figure legend, Results and Discussion sections related to Fig. 4, which was incorrect. This has been corrected in both the PDF and HTML versions of the Article.

