# Supplementary material for: Single cell transcriptome analysis of developing arcuate nucleus neurons uncovers their key developmental regulators
Source: Nat Commun. 2019 Aug 16;10:3696. doi: 10.1038/s41467-019-11667-y (PMC6697706; doi:10.1038/s41467-019-11667-y)
Supplement: Supplementary file 1 — Supplementary Information [file 41467_2019_11667_MOESM1_ESM.pdf]

Supplementary information

Single cell transcriptome analysis of developing arcuate nucleus neurons uncovers their key developmental regulators

Christian Huisman, Hyeyoung Cho, Olivier Brock, Su Jeong Lim, Sung Min Youn, Younjung Park, Sangsoo Kim, Soo-Kyung Lee, Alessio Delogu, and Jae W. Lee

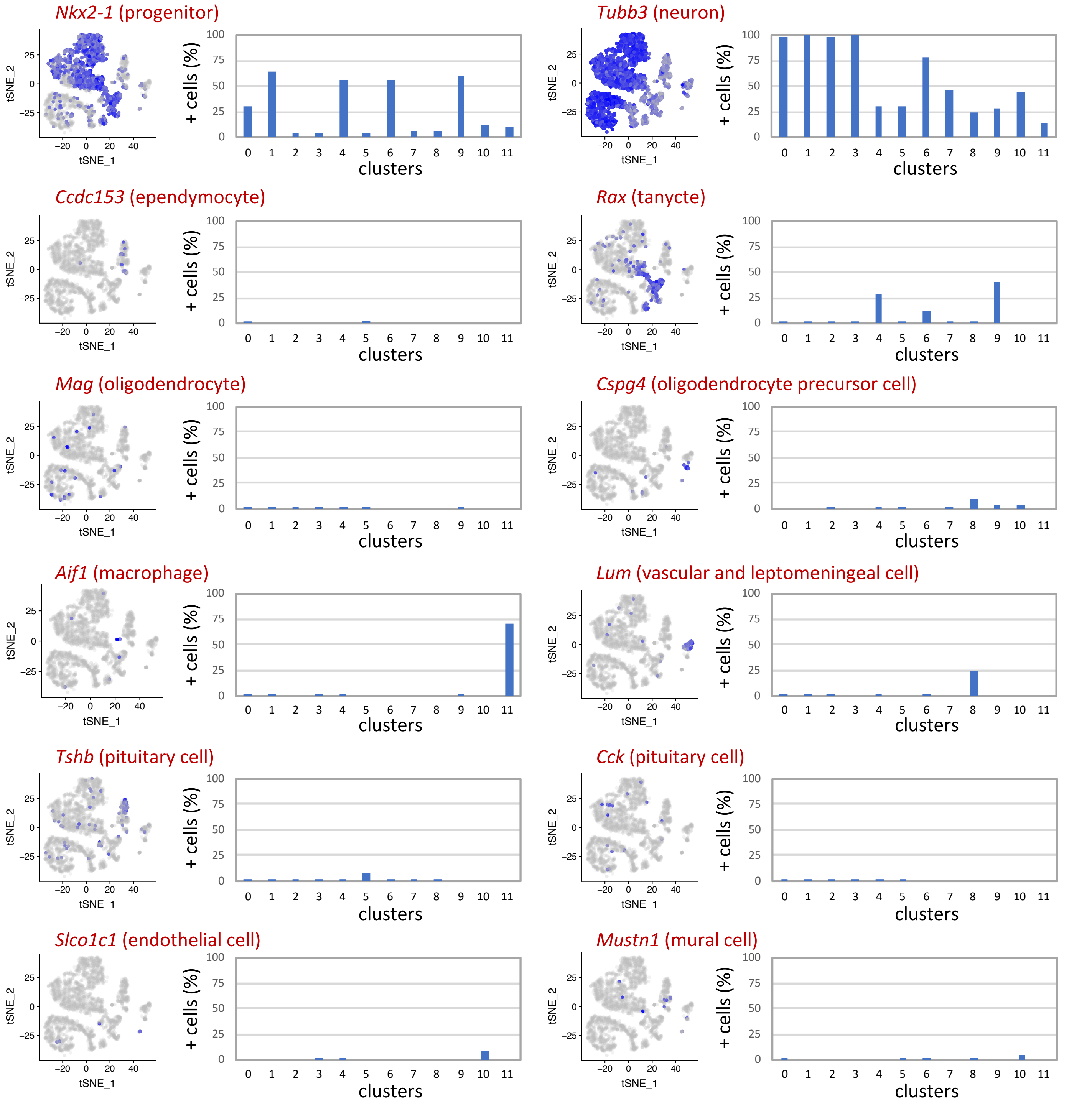

Supplementary Fig. 1. Cellular identity of each of the initial 12 clusters was determined by examining the expression level of key markers of different cell types in spectral tSNE plot and a graph for % of cells expressing the marker gene in each cluster. Overall, a majority of cells (78-100%) in the clusters 0, 1, 2, 3 and 6 as well as 15-46% of cells in the remaining clusters expressed the neuronal marker gene *Tubb3*. Also 31-65% of cells in the clusters 0, 1, 4, 6 and 9 still express the progenitor marker *Nkx2-1*. Cells in the cluster 11 appear to be macrophages based on the expression of the macrophage marker gene *Aif1* in 70% of cells in this cluster. Interestingly, the clusters 4 and 9 express both the neuronal marker gene *Tubb3* and the tanycyte marker gene *Rax*, while the cluster 8 express both the neuronal marker gene *Tubb3* and the vascular and leptomeningeal cell marker gene *Lum*, raising the interesting possibility that these non-neuronal cell types may also arise from *Nkx2-1*$^{+}$ progenitors. Most non-neuronal marker genes that we tested were not well expressed in the clusters c0-c11.

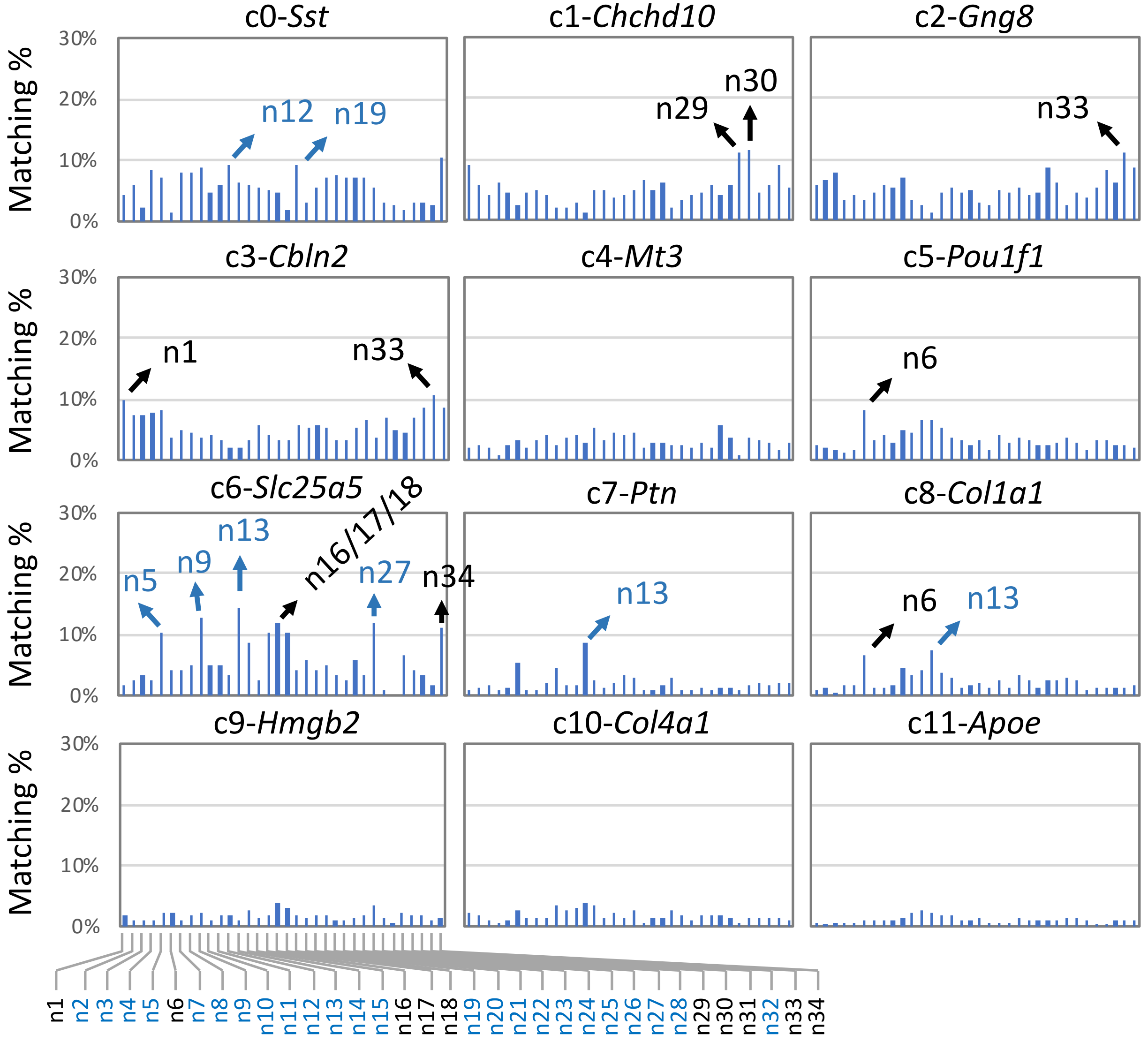

Supplementary Fig. 2. Similarity of the E15 clusters c0 to c11 to the adult neurons n1 to n34 (which include 24 ARC neurons highlighted in blue) was determined by calculating what % of genes specifically enriched in each cluster belong to the top 200 genes enriched in each of the adult neurons n1 to n34.

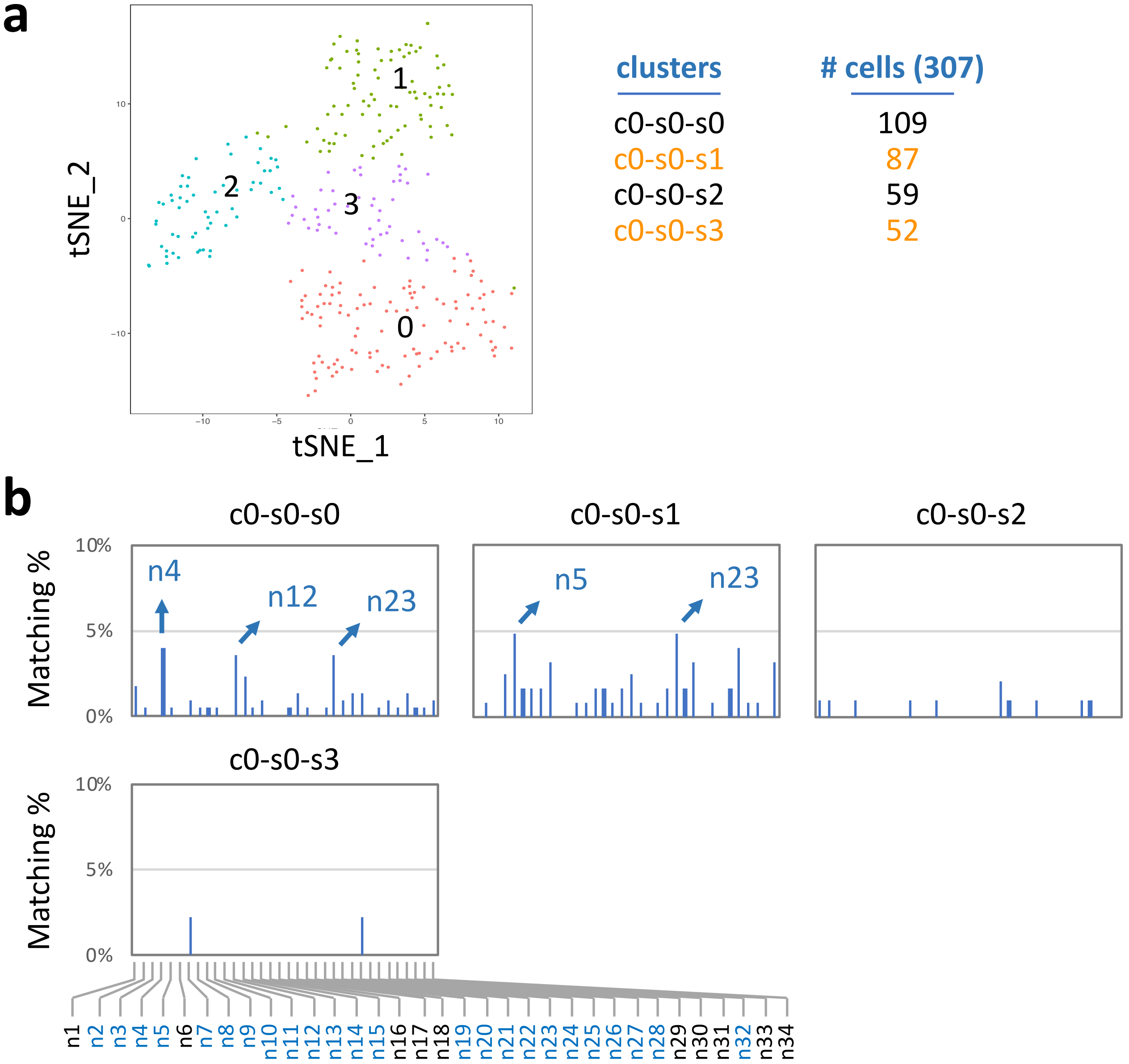

Supplementary Fig. 3. Similarity of the E15 subclusters c0-s0-s0 to c0-s0-s3 to the adult neurons n1 to n34 (which include 24 ARC neurons highlighted in blue) was determined by calculating what % of genes enriched in each subcluster belong to the top 50 genes enriched in each of the adult neurons n1 to n34. The adult ARC neurons n4, n12, n23 are highly related to each other (see text) and the initial match of the subcluster c0-s0 to these three types of neurons (Fig. 2) were still maintained in the subcluster c0-s0-s0, suggesting that the cells in the original subcluster c0-s0 may develop to become n4, n12, n23 ARC neurons in adult ARC.

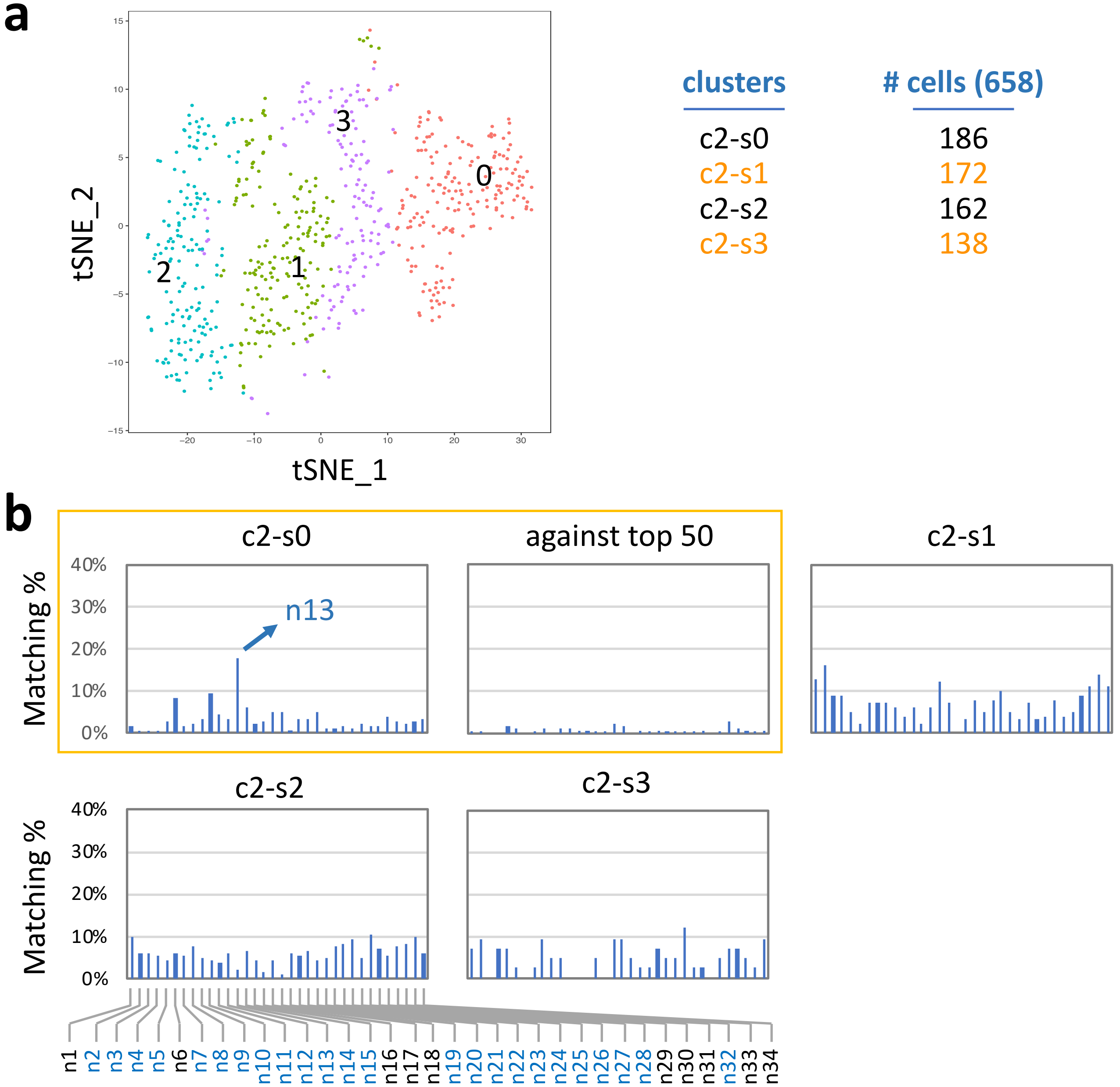

Supplementary Fig. 4. Similarity of the E15 subclusters c2-s0 to c2-s3 to the adult neurons n1 to n34 (which include 24 ARC neurons highlighted in blue) was determined by calculating what% of genes specifically enriched in each subcluster belong to the top 200 genes enriched in each of the adult neurons n1 to n34. While the subcluster c2-s0 showed similarity to n13 $Agrp^{Gm8773}$-neurons, the similarity was lost when the analysis was repeated against the top 50 genes enriched in each adult neuron, n1 to n34. Because expression of the top 50 adult ARC neuronal genes in developing neurons represents more advanced development, the re-analysis against the top 50 adult neuronal genes led us to predict that cells in the subcluster c2-s0, while they appear to be still in an early stage of development, may eventually develop to become n13 $Agrp^{Gm8773}$-neurons in adult ARC.

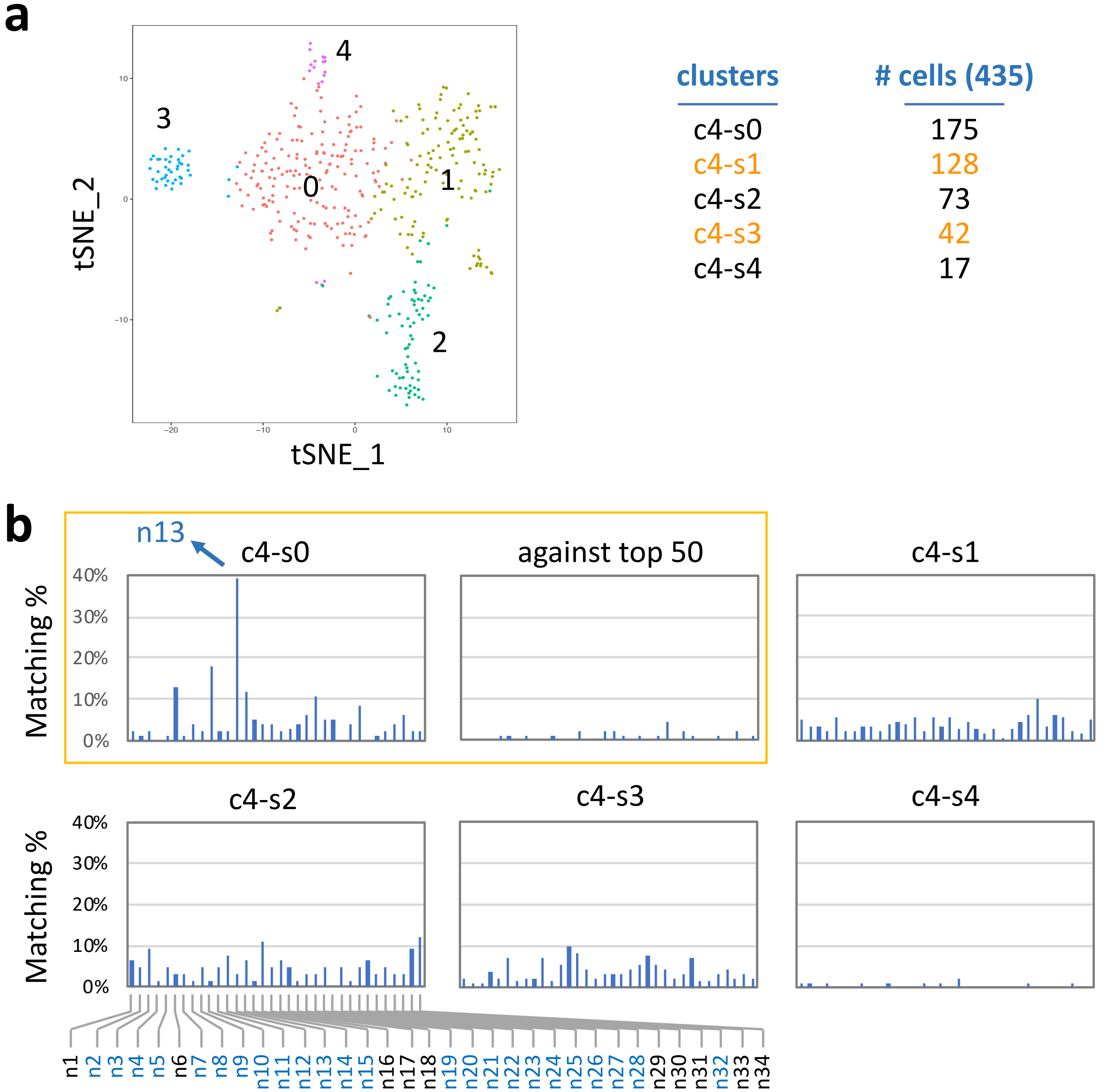

Supplementary Fig. 5. Similarity of the E15 subclusters c4-s0 to c4-s4 to the adult neurons n1 to n34 (which include 24 ARC neurons highlighted in blue) was determined by calculating what% of genes specifically enriched in each subcluster belong to the top 200 genes enriched in each of the adult neurons n1 to n34. While the subcluster c4-s0 showed similarity to n13 Agrp^{Gm8773}-neurons, it lost the similarity when the analysis was repeated against the top 50 genes enriched in each of the adult neurons n1 to n34. Thus, it is possible that cells in the subcluster c4-s0 may represent early precursors to n13 Agrp^{Gm8773}-neurons.

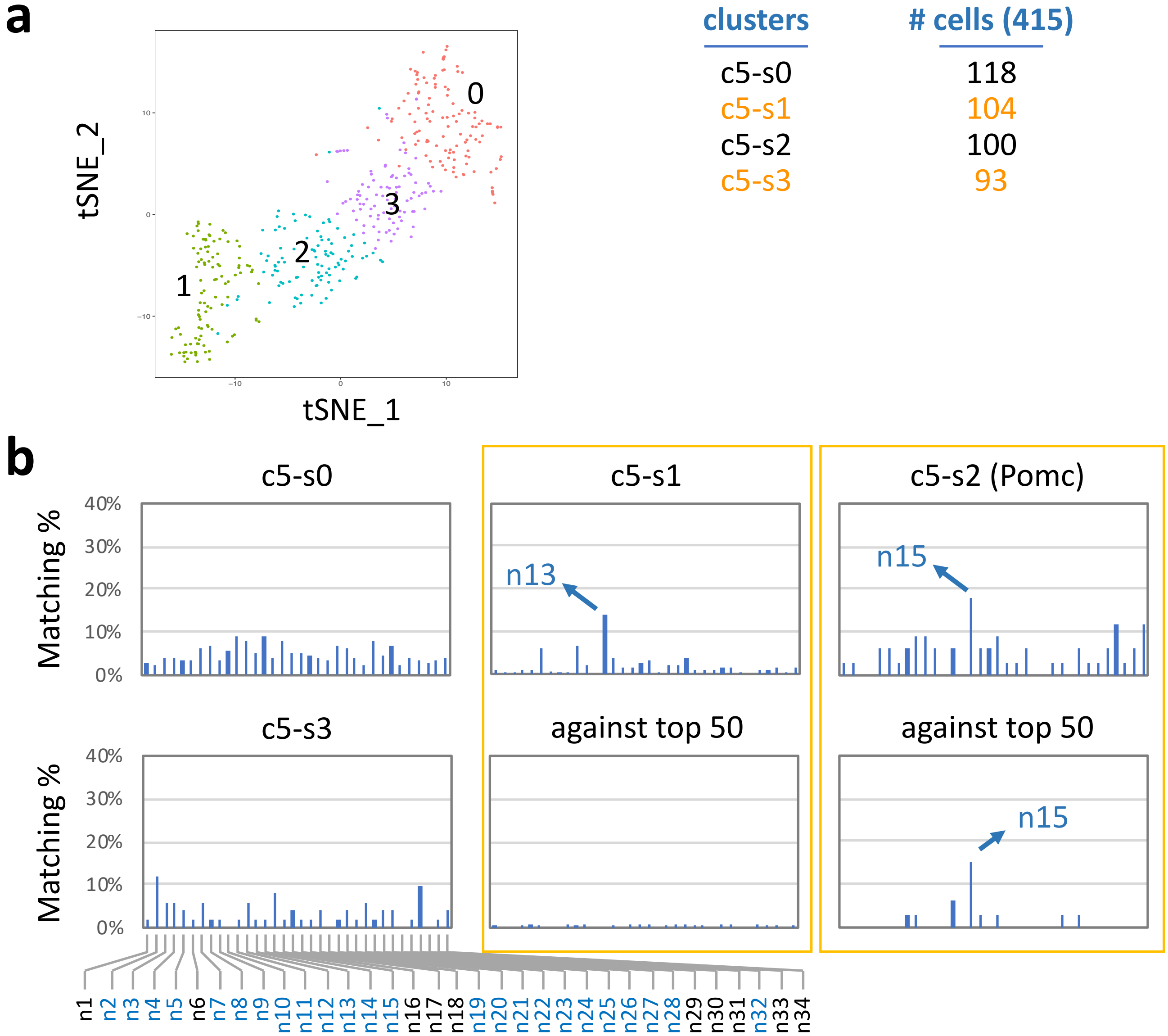

Supplementary Fig. 6. Similarity of the E15 subclusters c5-s0 to c5-s3 to the adult neurons n1 to n34 (which include 24 ARC neurons highlighted in blue) was determined by calculating what %of genes specifically enriched in each subcluster belong to the top 200 genes enriched in each of the adult neurons n1 to n34. While the subcluster c5-s1 showed similarity to n13 $Agrp^{Gm8773}$-neurons, it lost the similarity when the analysis was repeated against the top 50 genes enriched in each of the adult neurons n1 to n34. Thus, it is possible that cells in the subcluster c5-s1 may eventually develop to n13 $Agrp^{Gm8773}$-neurons. Also, cells in the subcluster c5-s2 showed similarity to n15 $Pomc^{Anxa2}$-neurons, which was still maintained when the analysis was performed against the top 50 genes enriched in each of the adult neurons n1 to n34. Thus, cells in the subcluster c5-s2 are likely to develop to become n15 $Pomc^{Anxa2}$-neurons in adult ARC.

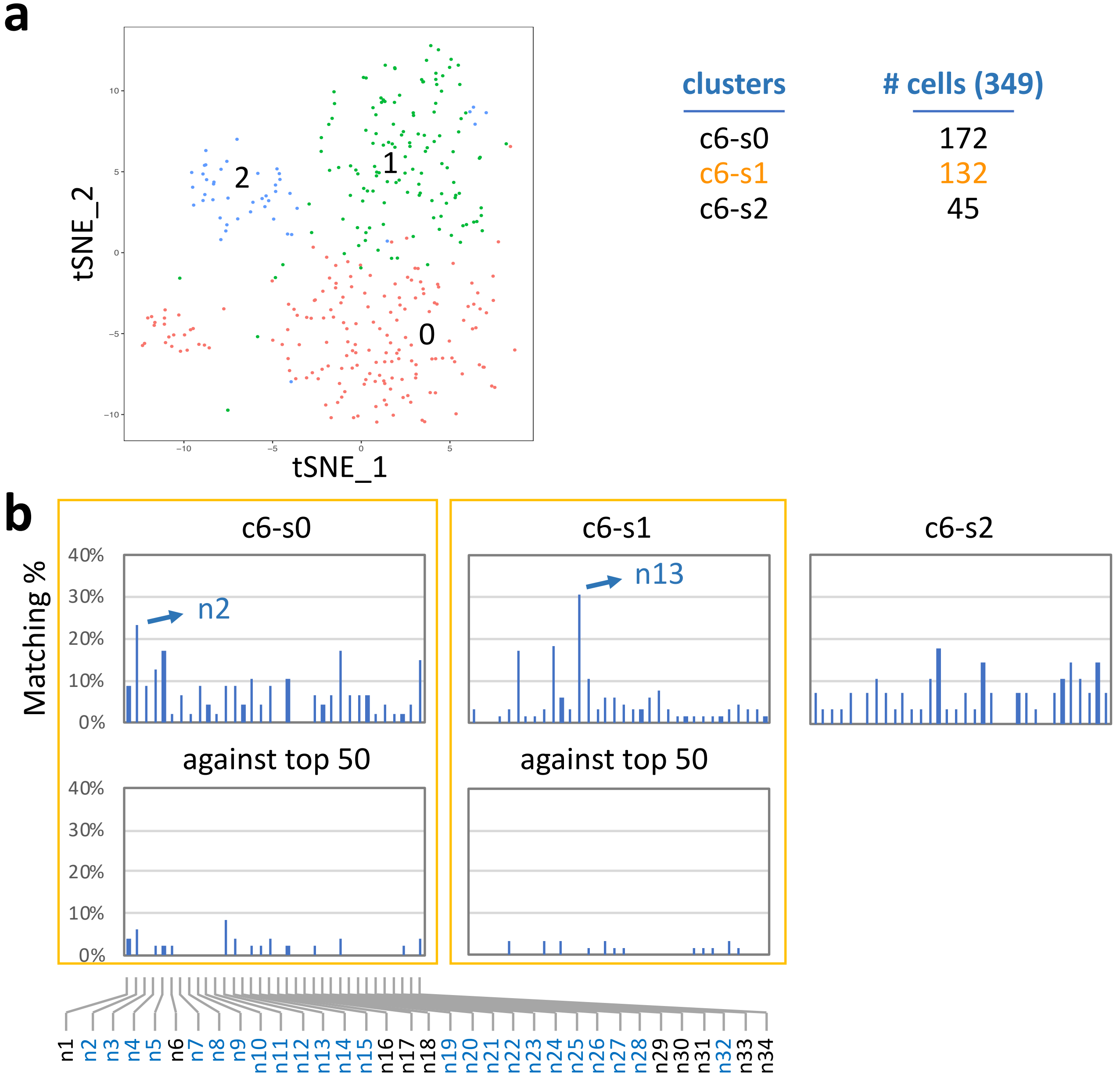

Supplementary Fig. 7. Similarity of the E15 subclusters c6-s0 to c6-s2 to the adult neurons n1 to n34 (which include 24 ARC neurons highlighted in blue) was determined by calculating what% of genes specifically enriched in each subcluster belong to the top 200 genes enriched in each of the adult neurons n1 to n34. Cells in the subclusters c6-s0 and c6-s1 showed similarity to n2 and n13 $Agrp^{Gm8773}$-neurons, respectively. However, the similarity was lost when the analysis was repeated against the top 50 genes enriched in each of the adult neurons n1 to n34. Thus, it is possible that cells in these two subclusters may represent early precursors to n2 and n13 $Agrp^{Gm8773}$-neurons, respectively.

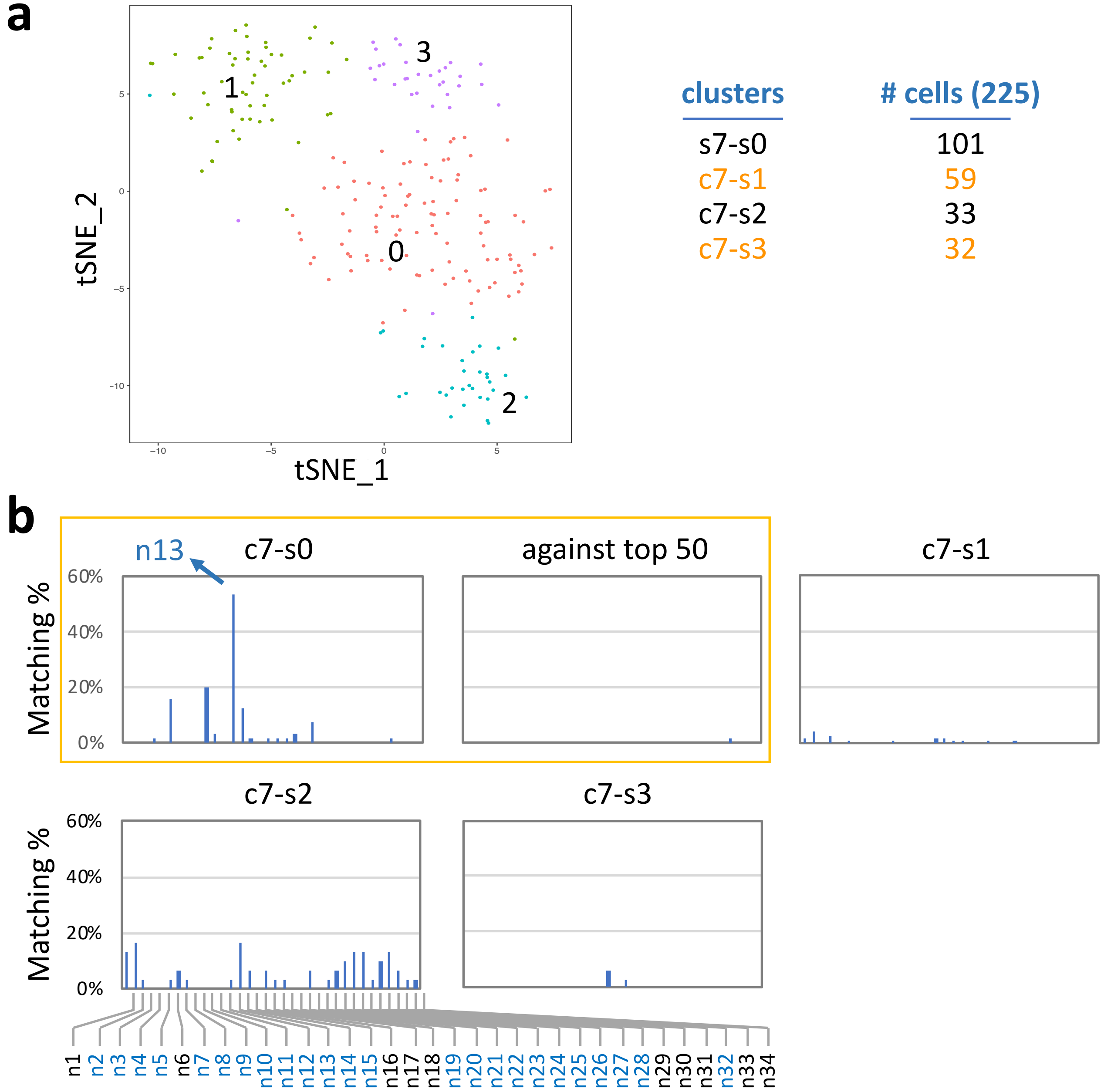

Supplementary Fig. 8. Similarity of the E15 subclusters c7-s0 to c7-s3 to the adult neurons n1 to n34 (which include 24 ARC neurons highlighted in blue) was determined by calculating what% of genes specifically enriched in each subcluster belong to the top 200 genes enriched in each of the adult neurons n1 to n34. While cells in the subcluster c7-s0 showed similarity to n13 Agrp^{Gm8773}-neurons, the similarity was lost when the analysis was repeated against the top 50 genes enriched in each of the adult neurons n1 to n34. These results suggest that cells in the subcluster c7-s0 may represent early precursors to n13 Agrp^{Gm8773}-neurons.

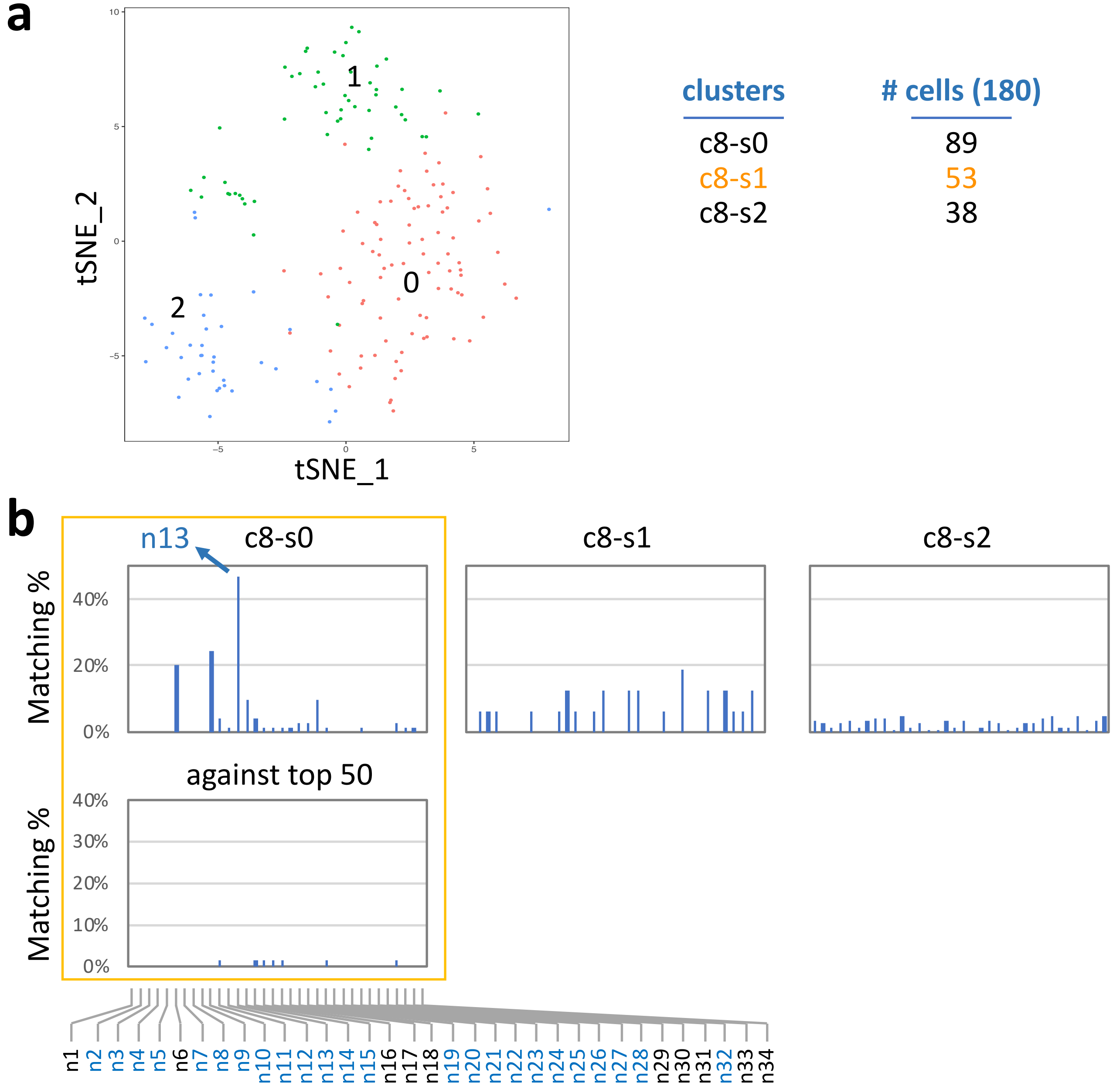

Supplementary Fig. 9. Similarity of the E15 subclusters c8-s0 to c8-s2 to the adult neurons n1 to n34 (which include 24 ARC neurons highlighted in blue) was determined by calculating what% of genes specifically enriched in each subcluster belong to the top 200 genes enriched in each of the adult neurons n1 to n34. While cells in the subcluster c8-s0 showed highest similarity to n13 Agrp^{Gm8773}-neurons, the similarity was lost when the analysis was repeated against the top 50 genes enriched in each of the adult neurons n1 to n34. These results suggest that the cells in the subcluster c8-s0 may represent early precursors to n13 Agrp^{Gm8773}-neurons.

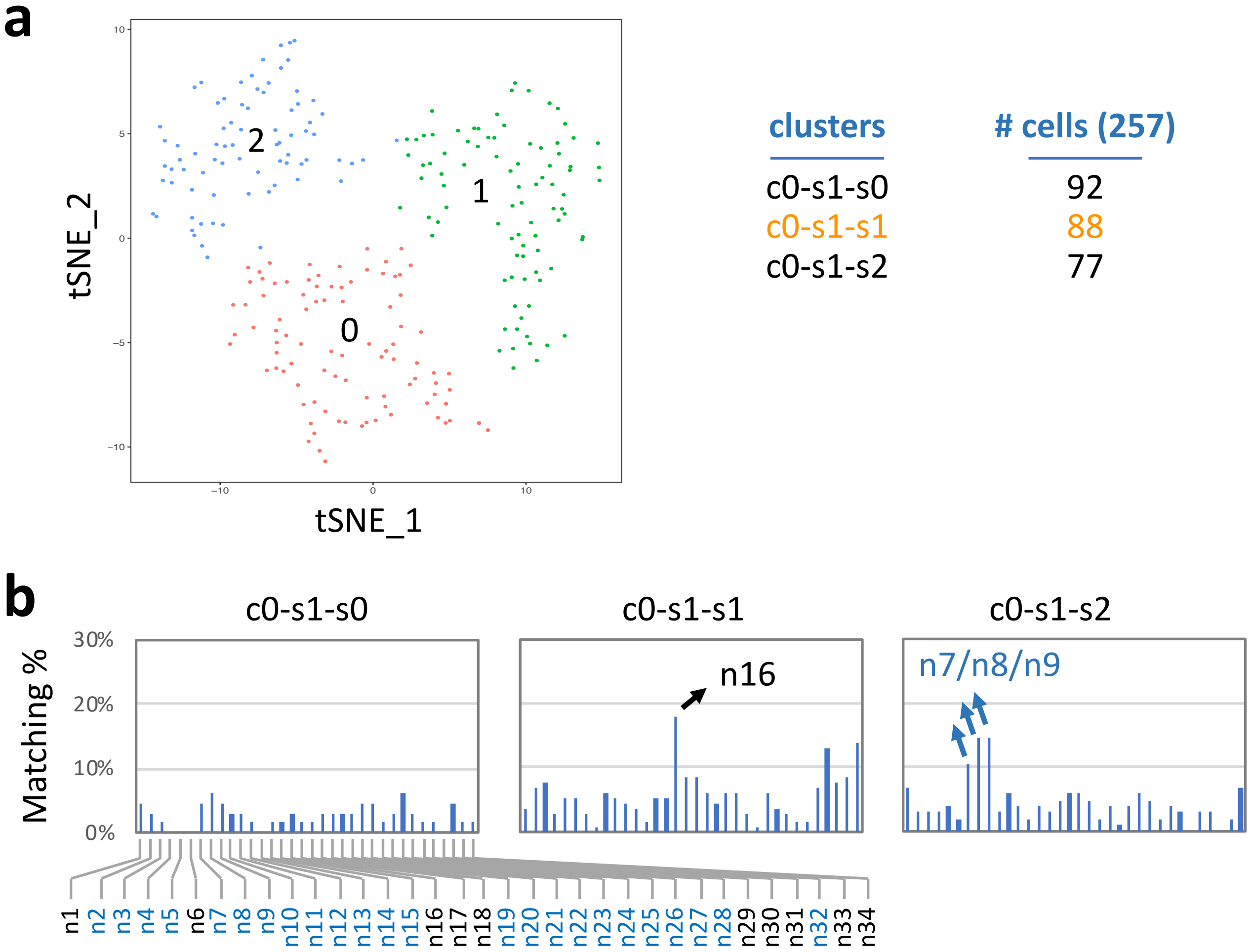

Supplementary Fig. 10. Similarity of the E15 subclusters c0-s1-s0 to c0-s1-s2 to the adult neurons n1 to n34 (which include 24 ARC neurons highlighted in blue) was determined by calculating what % of genes enriched in each subcluster belong to the top 200 genes enriched in each of the adult neurons n1 to n34. Cells in the subclusters c0-s1-s1 and c0-s1-s2 showed similarity to the adult n16 non-ARC neurons and n7/n8/n9 ARC neurons, respectively, suggesting that the original subcluster c0-s1 represents a pool of these neurons. These results suggest that the subcluster c0-s1-s2 may eventually segregate and develop to become n7/n8/n9 neurons, respectively, in adult ARC. These three types of ARC neurons share some key features (see text).

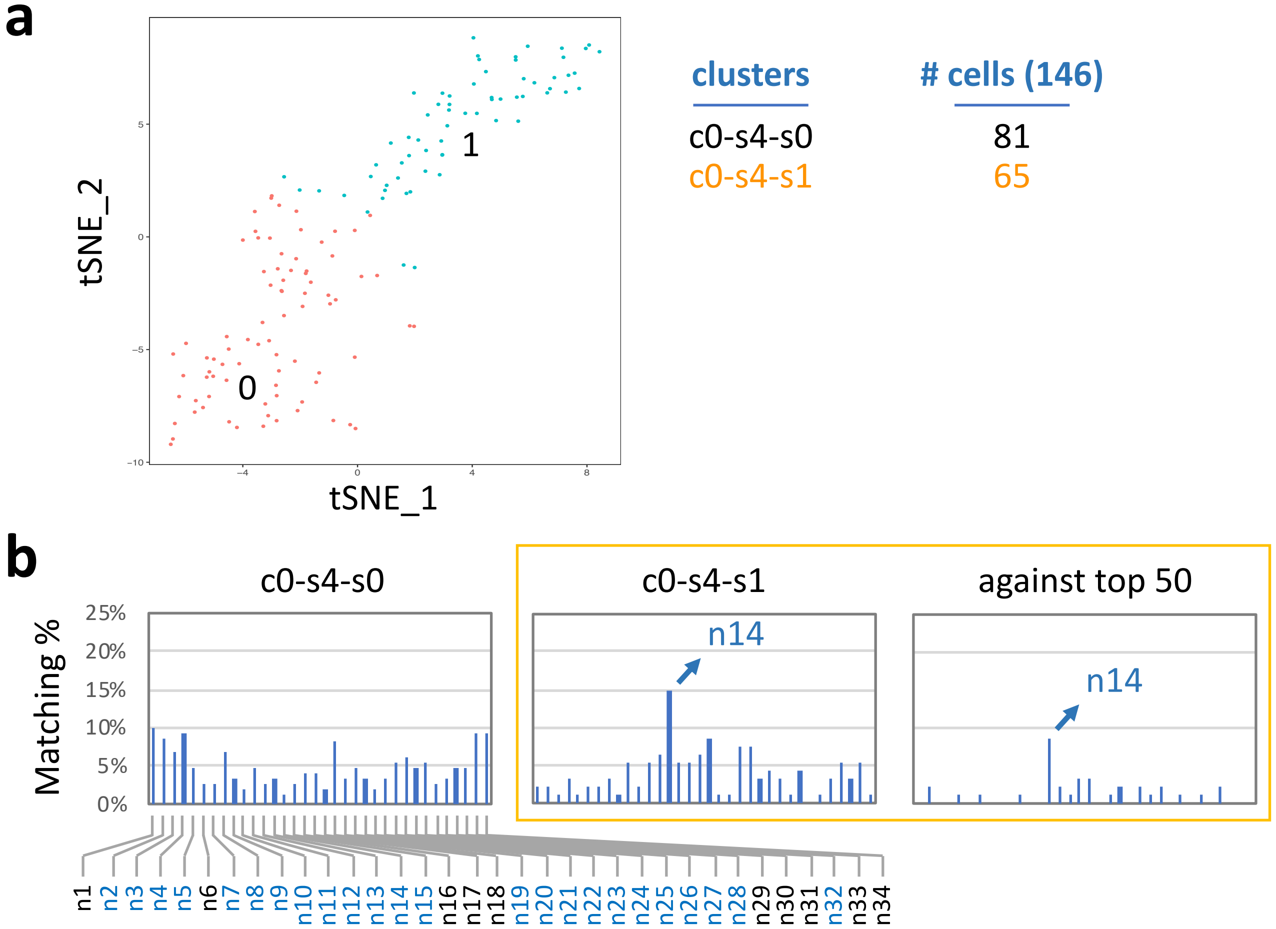

Supplementary Fig. 11. Similarity of the E15 subclusters c0-s4-s0 to c0-s4-s1 to the adult neurons n1 to n34 (which include 24 ARC neurons highlighted in blue) was determined by calculating what % of genes enriched in each subcluster belong to the top 200 genes enriched in each of the adult neurons n1 to n34. Cells in the subcluster c0-s4-s1 showed similarity to n14 Pomc^{Ttr}-neurons, which was still maintained when the analysis was repeated against the top 50 genes enriched in each of the adult neurons n1 to n34. These results suggest that cells in the subcluster c0-s4-s1 are likely to develop to become n14 Pomc^{Ttr}-neurons in adult ARC.

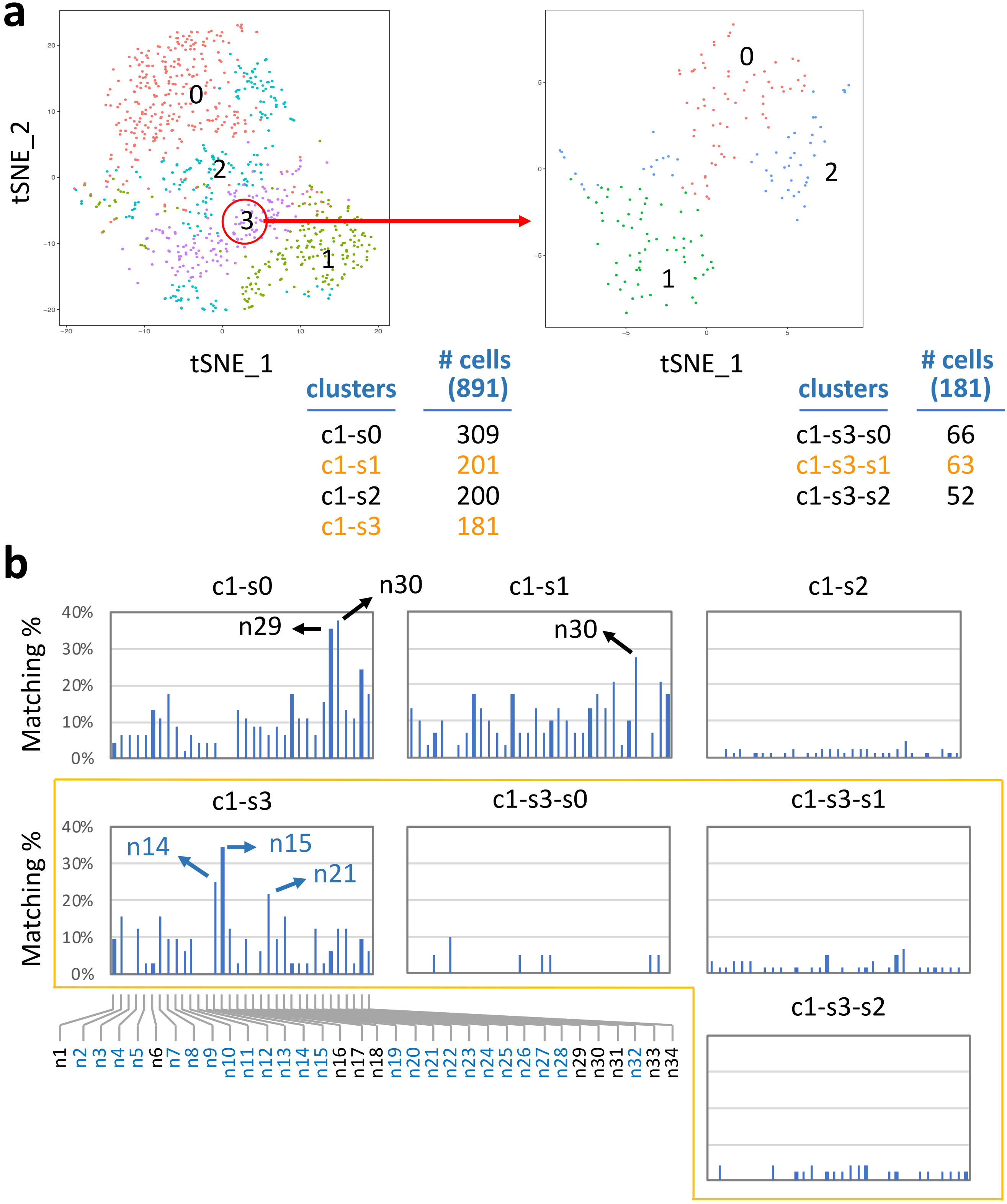

Supplementary Fig. 12. Similarity of the E15 subclusters c1-s0 to c1-s3 to the adult neurons n1 to n34 (which include 24 ARC neurons highlighted in blue) was determined by calculating what % of genes specifically enriched in each subcluster belong to the top 200 genes enriched in each of the adult neurons n1 to n34. Given that cells in the subcluster c1-s3 showed similarity to n14, n15 and n21 Pomc-neurons, it was further reclustered to 3 subclusters. However, none of them showed similarity to n14, n15 and n21 Pomc-neurons, suggesting that the subcluster c1-s3 may represent early precursors to all three types of Pomc-neurons.

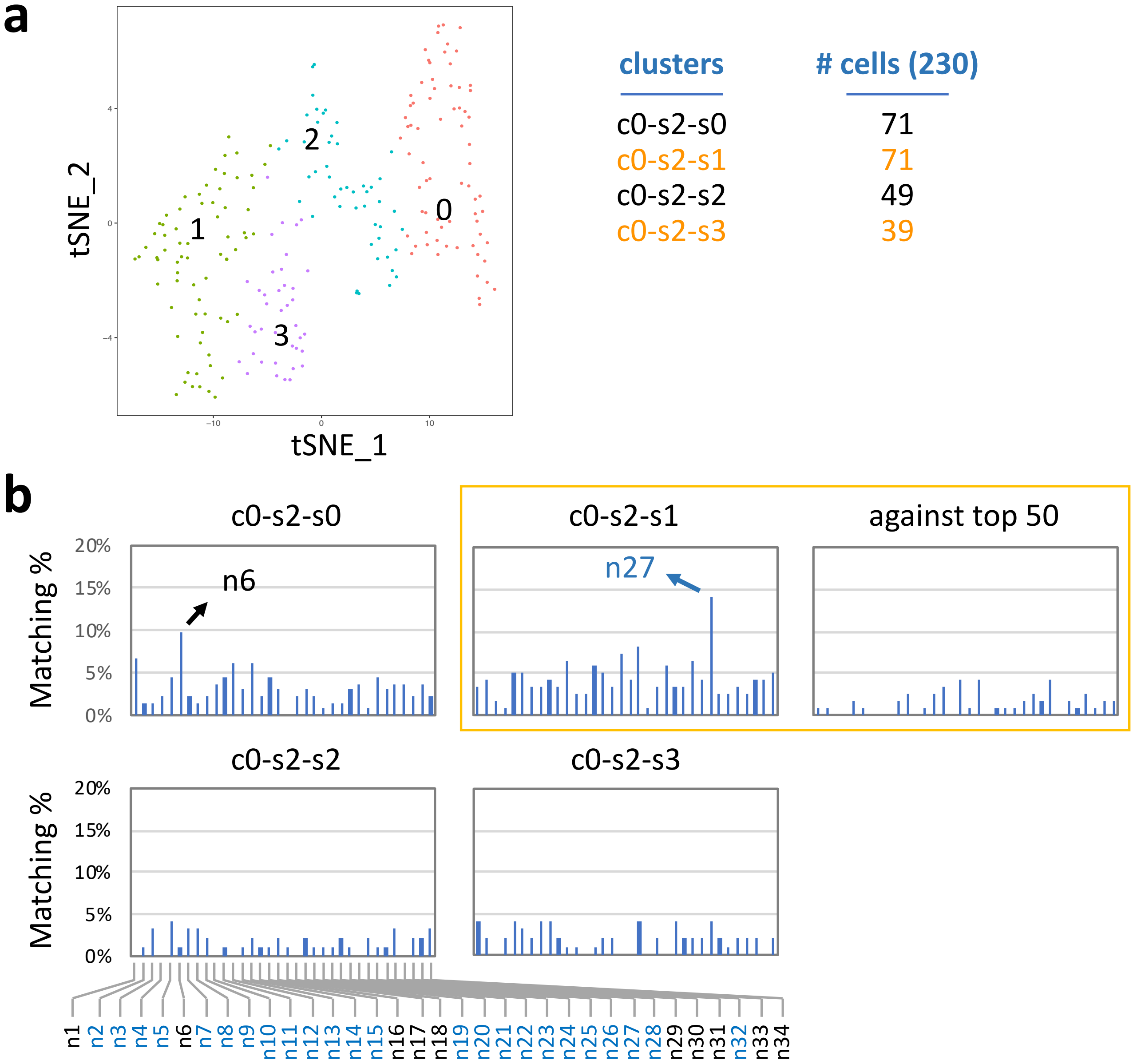

Supplementary Fig. 13. Similarity of the E15 subclusters c0-s2-s0 to c0-s2-s3 to the adult neurons n1 to n34 (which include 24 ARC neurons highlighted in blue) was determined by calculating what % of genes enriched in each subcluster belong to the top 200 genes enriched in each of the adult neurons n1 to n34. The sublcuster c0-s2-s1 matches one of the original ARC neuronal types identified in the sublcuster c0-s2 (n27). However, the match to n27 was lost when the identity was determined against the top 50 genes enriched in each of the adult neurons n1 to n34. These results suggest that cells in the subcluster c0-s2-s1 may develop to become n27 ARC neurons in adult ARC (i.e., they could be early precursors to n27 neurons).

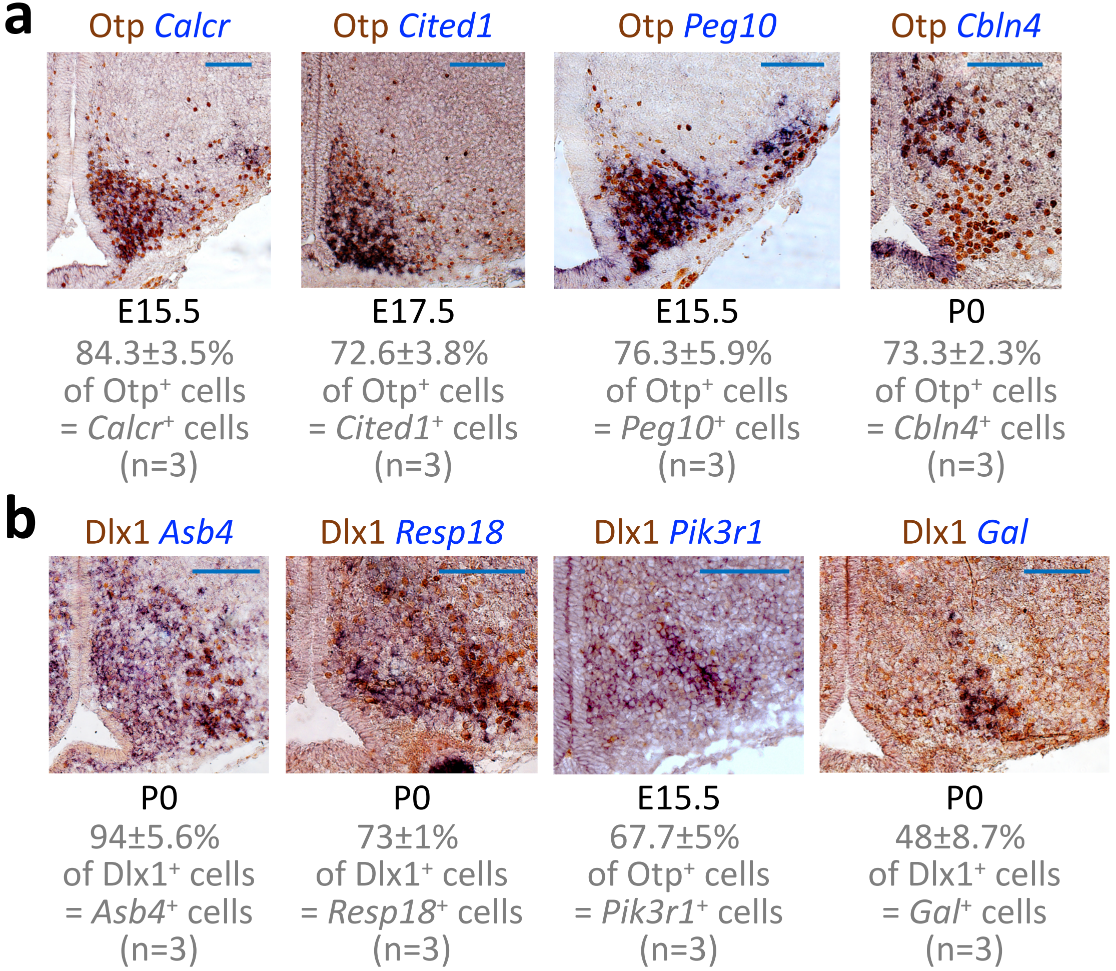

Supplementary Fig. 14. Validation of our E15 scRNA-seq results (I). **a** Using a combination of IHC and ISH, we confirmed co-expression of *Calcr*, *Cited1*, *Peg10*, and *Cbln4* (ISH, blue) in cells expressing Otp (IHC, brown) in the ARC at indicated developmental stages. Otp was used as a marker for Agrp-neurons (Otp also labels non-Agrp-neurons). **b** We also found co-expression of *Asb4*, *Resp18*, *Pik3r1*, and *Gal* (ISH, blue) in cells expressing Dlx1 (a marker for Ghrh- and other Th-neurons in the ARC; IHC, brown). All scale bars are for 100 μm.

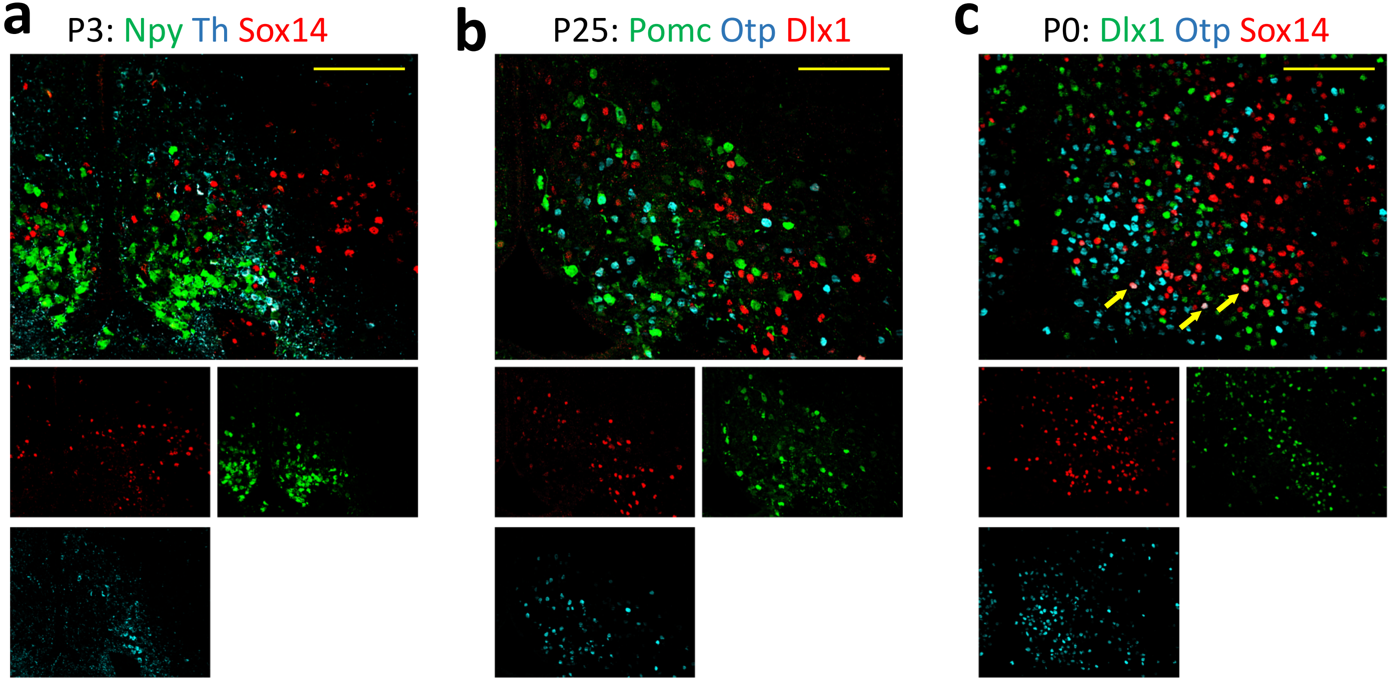

Supplementary Fig. 15. Validation of our E15 scRNA-seq results (II). Using IHC, we confirmed segregation of multiple cell types that we identified in our scRNAseq analysis of E15 ARC. Npy and Pomc (green) signals are from *Npy-Gfp* (**a**) and *Pomc-eGfp* (**b**) reporter mice, respectively. Signals for Npy, Th and Sox14 (**a**), Pomc, Otp and Dlx1 (**b**), and Dlx1, Otp and Sox14 (**c**) in the ARC, except a few cells that coexpress Sox14 and Otp (yellow arrows) **(c),** do not overlap, suggesting: Npy^+ Agrp-neurons, Th^+ neurons (which include Ghrh-neurons) and $Sox14^+$ Kisspeptin-neurons represent distinct populations of neurons (**a**); $Pomc^+$ Pomc-neurons, Otp^+ neurons (such as Agrp-neurons) and $Dlx1^+$ neurons (e.g., Ghrh-neurons) represent distinct populations of neurons (**b**); and $Dlx1^+$ neurons, Otp^+ neurons and $Sox14^+$ Kisspeptin-neurons represent mostly distinct populations of neurons (**c**). All scale bars are for 100 μm.

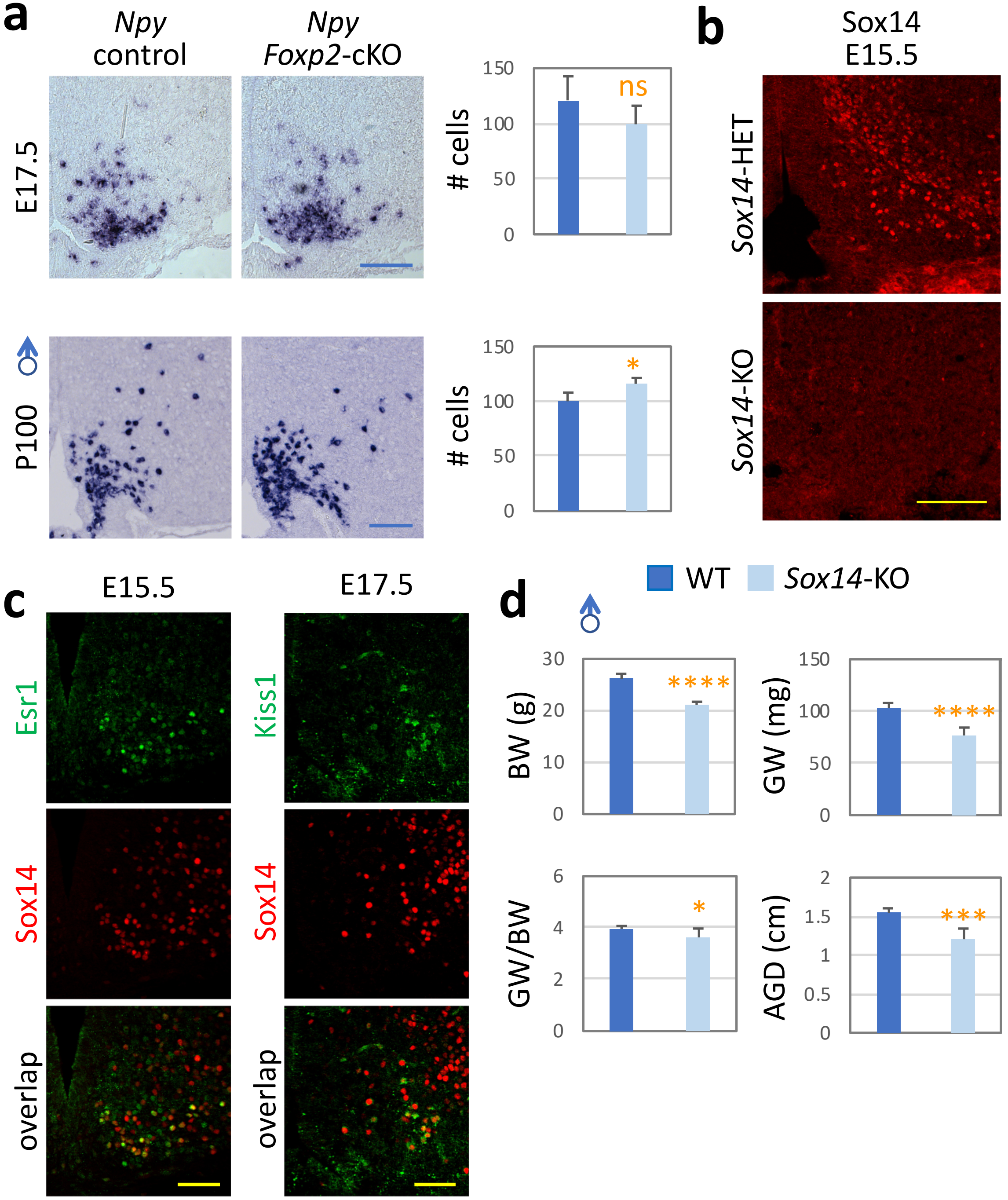

Supplementary Fig. 16. a ISH against *Npy* reveals that the number of *Npy*$^{+}$ cells (i.e., Agrp-neurons) in the ARC is not reduced in *Foxp2*-cKO mice relative to control mice at both E17.5 and P100 (male). **b** Our home-made Sox14 antibody was tested for specificity using E15.5 *Sox14*-HET vs. *Sox14*-KO ARC. **c** IHC with antibodies against Sox14 (red) and Esr1 or Kiss1 (green) reveals co-expression of Sox14 with Esr1 and Kiss1 in a subset of cells, which likely represent Kisspeptin-neurons. **d** Measurement of body weight, GW (for gonad weight), and AGD (for anogenital distance) for P100 male mice. The number of mice per each genotype was 3~9. All scale bars are for 100 μm. Statistical differences were determined by Student's t-test; $p < 0.05$ (one asterisk), $p < 0.001$ (three asterisks), $p < 0.0001$ (four asterisks) and 'ns' indicates not-significant ($p > 0.05$).
