# Supplementary material for: Single cell transcriptome analysis of developing arcuate nucleus neurons uncovers their key developmental regulators
Source: Nat Commun. 2019 Aug 16;10:3696. doi: 10.1038/s41467-019-11667-y (PMC6697706; doi:10.1038/s41467-019-11667-y)
Supplement: Supplementary file 3 — Description of Additional Supplementary Files [file 41467_2019_11667_MOESM3_ESM.docx]

**Description of Additional Supplementary Files**

File Name: Supplementary Data 1
Description: Lists of specifically enriched genes in c0-c11 clusters

File Name: Supplementary Data 2
Description: Lists of specifically enriched genes in c0-s0 to c0-s6 subclusters

File Name: Supplementary Data 3
Description: Lists of specifically enriched genes in other subclusters
